# Investigation of candidate genes and mechanisms underlying obesity associated type 2 diabetes mellitus using bioinformatics analysis and screening of small drug molecules

**DOI:** 10.1186/s12902-021-00718-5

**Published:** 2021-04-26

**Authors:** G. Prashanth, Basavaraj Vastrad, Anandkumar Tengli, Chanabasayya Vastrad, Iranna Kotturshetti

**Affiliations:** 1 Department of General Medicine, Basaveshwara Medical College, Chitradurga, Karnataka 577501 India; 2 Department of Biochemistry, Basaveshwar College of Pharmacy, Gadag, Karnataka 582103 India; 3 Department of Pharmaceutical Chemistry, JSS College of Pharmacy, Mysuru and JSS Academy of Higher Education & Research, Mysuru, Karnataka 570015 India; 4 Biostatistics and Bioinformatics, Chanabasava Nilaya, Bharthinagar, Dharwad, Karnataka 580001 India; 5 Department of Ayurveda, Rajiv Gandhi Education Society`s Ayurvedic Medical College, Ron, Karnataka 582209 India

**Keywords:** obesity associated type 2 diabetes mellitus, differentially expressed gene, pathway, protein-protein interaction network, miRNA-target genes regulatory network

## Abstract

**Background:**

Obesity associated type 2 diabetes mellitus is a metabolic disorder ; however, the etiology of obesity associated type 2 diabetes mellitus remains largely unknown. There is an urgent need to further broaden the understanding of the molecular mechanism associated in obesity associated type 2 diabetes mellitus.

**Methods:**

To screen the differentially expressed genes (DEGs) that might play essential roles in obesity associated type 2 diabetes mellitus, the publicly available expression profiling by high throughput sequencing data (GSE143319) was downloaded and screened for DEGs. Then, Gene Ontology (GO) and REACTOME pathway enrichment analysis were performed. The protein - protein interaction network, miRNA - target genes regulatory network and TF-target gene regulatory network were constructed and analyzed for identification of hub and target genes. The hub genes were validated by receiver operating characteristic (ROC) curve analysis and RT- PCR analysis. Finally, a molecular docking study was performed on over expressed proteins to predict the target small drug molecules.

**Results:**

A total of 820 DEGs were identified between healthy obese and metabolically unhealthy obese, among 409 up regulated and 411 down regulated genes. The GO enrichment analysis results showed that these DEGs were significantly enriched in ion transmembrane transport, intrinsic component of plasma membrane, transferase activity, transferring phosphorus-containing groups, cell adhesion, integral component of plasma membrane and signaling receptor binding, whereas, the REACTOME pathway enrichment analysis results showed that these DEGs were significantly enriched in integration of energy metabolism and extracellular matrix organization. The hub genes CEBPD, TP73, ESR2, TAB1, MAP 3K5, FN1, UBD, RUNX1, PIK3R2 and TNF, which might play an essential role in obesity associated type 2 diabetes mellitus was further screened.

**Conclusions:**

The present study could deepen the understanding of the molecular mechanism of obesity associated type 2 diabetes mellitus, which could be useful in developing therapeutic targets for obesity associated type 2 diabetes mellitus.

## Introduction

Obesity associated type 2 diabetes is one of the most common metabolic disorder worldwide [1]. Type 2 diabetes mellitus is characterized by insulin deficiency due to pancreatic β-cell inactivation and insulin resistance [2]. Genetic factors, hyperinsulinemia, atherogenic dyslipidemia, glucose intolerance, hypertension, prothrombic state, hyperuricemia and polycystic ovary syndrome are the key risk factors for the occurrence and progression of type 2 diabetes mellitus [3]. Obesity associated type 2 diabetes mellitus affects the vital organs such as heart [4], brain [5], kidney [6] and eye [7]. Etiology and advancement of obesity associated type 2 diabetes mellitus is more complex and still understandable. Therefore, it is essential to understand the precise molecular mechanisms associated in the progression of obesity associated type 2 diabetes mellitus and thus to establish valid diagnostic and therapeutic strategies.

Current evidence has shown that genetic predisposition plays a key role in the advancement of obesity associated type 2 diabetes mellitus [8]. Recently, several genes and pathways have been found to participate in the occurrence and advancement of obesity associated type 2 diabetes mellitus [9], including FGF21 [10], pro-opiomelanocortin (POMC) [11], PI3K/AKT pathway [12] and JAK/STAT pathway [13]. However, the current knowledge is insufficient to explain and understand how these crucial genes and signaling pathways are associated with advancement of obesity associated type 2 diabetes mellitus. Therefore, there is a great need to find new prognostic and diagnostics biomarkers, and to advance novel techniques to enlighten the molecular mechanisms controlling the progression of obesity associated type 2 diabetes mellitus.

Bioinformatics analysis of expression profiling by high throughput sequencing data has shown great promise to discover potential key genes and signaling pathways with significant roles in metabolic disorder [14], to identify new prognostic and diagnostics biomarkers, and biological processes implicated in obesity associated type 2 diabetes mellitus. In this investigation, using bioinformatics analysis, we aimed to investigate expression profiling by high throughput sequencing data to determine differentially expressed genes (DEGs) and significant pathways in obesity associated type 2 diabetes mellitus. After searching the Gene Expression Omnibus (GEO) database [15], we selected RNA sequencing dataset GSE143319 for identifying DEGs for obesity associated type 2 diabetes mellitus. This dataset gives more information about obesity associated type 2 diabetes mellitus elevates patient’s risk of nonalcoholic steatohepatitis (NASH), cardiovascular disease and cancer. Gene Ontology (GO) and pathway enrichment analysis were performed. A hub and target genes were identified from protein-protein interaction (PPI) network, modules, miRNA-target genes regulatory network and TF-target gene regulatory network. Subsequently, hub genes were validated by using receiver operating characteristic (ROC) curve and RT- PCR analysis. Finally, molecular docking studies performed for prediction of small drug molecules.

## Materials and Methods

### RNA sequencing data

The expression profiling by high throughput sequencing dataset GSE143319 deposited by Ding et al [16] into the GEO database were obtained on the GPL20301 platform (Illumina HiSeq 4000 (Homo sapiens)). This dataset is provided for 30 samples, including 15 samples of metabolically healthy obese and 15 samples of a metabolically unhealthy obese.

### Identification of DEGs

The limma [17] in R bioconductor package was utilized to screen DEGs between metabolically healthy obese and metabolically unhealthy obese. These DEGs were identified as important genes that might play an important role in the development of obesity associated type 2 diabetes mellitus. The cutoff criterion were ∣log fold change (FC)∣ > 0.2587 for up regulated genes, ∣log fold change (FC)∣ < -0.2825 for down regulated genes and adjusted P value < 0.05.

### GO and pathway enrichment analyses

ToppGene (ToppFun) (https://toppgene.cchmc.org/enrichment.jsp) [18], which is a useful online database that integrates biologic data and provides a comprehensive set of functional annotation information of genes as well as proteins for users to analyze the functions or signaling pathways. GO (https://geneontology.org/) [19] enrichment analysis (biologic processes [BP], cellular components [CC], and molecular functions [MF]) is a strong bioinformatics tool to analyze and annotate genes. The REACTOME (https://reactome.org/) [20] is a pathway database resource for understanding high-level gene functions and linking genomic information from large scale molecular datasets. To analyze the function of the DEGs, biologic analyses were performed using GO and REACTOME pathway enrichment analysis via ToppGene online database.

### PPI network construction and module analysis

IMEX interactome (https://www.imexconsortium.org/) [21] online PPI database was using to identify the hub gene information in PPI network. Analyzing the interactions and functions of DEGs might give information about the controlling the progression of obesity associated type 2 diabetes mellitus. Cytoscape (version 3.8.2) (www.cytoscape.org) is a bioinformatics platform for constructing and visualizing PPI network [22]. Therefore, the topological properties includes node degree [23], betweenness centrality [24], stress centrality [25], closeness centrality [26] are analyzed in using Java plug-in Network Analyzer to obtain hub genes in the PPI network. The plug-in PEWCC1 of Cytoscape was applied to detect densely connected regions in PPI network. The significant modules in the PPI network was selected using PEWCC1 (https://apps.cytoscape.org/apps/PEWCC1) [27]. The criteria for selection were set as follows: Max depth = 100, degree cut-off = 2, node score cut-off = 0.2, PEWCC1 scores >5, and K-score = 2.

### Target gene – miRNA regulatory network construction and analysis

Obesity associated type 2 diabetes mellitus relating miRNAs and experimentally validated target genes were identified from miRNet database (https://www.mirnet.ca/) [28]. Obesity associated type 2 diabetes mellitus relating miRNAs and target genes were identified through target genes - miRNA regulatory network. Then the target genes - miRNA regulatory network was constructed and visualized by using Cytoscape software.

### Target gene – TF network regulatory construction and analysis

Obesity associated type 2 diabetes mellitus relating TFs and experimentally validated target genes were identified from TFs database NetworkAnalyst database (https://www.networkanalyst.ca/) [29]. Obesity associated type 2 diabetes mellitus relating TFs and target genes were identified through target genes - TF regulatory network. Then the target genes -TF regulatory network was constructed and visualized by using Cytoscape software.

### Receiver operating characteristic (ROC) curve analysis

The ROC curve was used to calculate classifiers in bioinformatics applications. To further assess the predictive accuracy of the hub genes, ROC analysis was performed to discriminate metabolically healthy obese from metabolically unhealthy obese. ROC curves for hub genes were generated using pROC in R [30] based on the obtained DEGs and their expression profiling by high throughput sequencing dataset. The area under the curve (AUC) was evaluated and used to compare the diagnostic value of hub genes.

### Validation of the expression levels of candidate genes by RT-PCR

Quantitative RT-PCR was conducted to validate the expressions of these hub genes in obesity associated type 2 diabetes mellitus. Total RNAs were extracted from Primary Subcutaneous Pre adipocytes; Normal Human cell line (ATCC® PCS-210-010™) and 3T3-L1 cells (ATCC® CL-173) using TRI Reagent® (Sigma, USA) according to instruction, followed by reverse transcription with Reverse transcription cDNA kit (Thermo Fisher Scientific, Waltham, MA, USA) and cDNA amplification through 7 Flex real-time PCR system (Thermo Fisher Scientific, Waltham, MA, USA). The expressions of hub genes were normalized to against beta actin expression. The data were calculated by the 2^−ΔΔCt^ method [31]. A primer used in the current investigation was listed in Table 1.

**Table 1:** The sequences of primers for quantitative RT-PCR

Genes	Forward Primers	Reverse Primers
CEBPD	CGGACTTGGTGCGTCTAAGATG	GCATTGGAGCGGTGAGTTTG
TP73	CCACCACTTTGAGGTCACTTT	CTTCAAGAGCGGGGAGTACG
ESR2	AGCACGGCTCCATATACATACC	TGGACCACTAAAGGAGAAAGGT
TAB1	AACTGCTTCCTGTATGGGGTC	AAGGCGTCGTCAATGGACTC
MAP 3K5	CTGCATTTTGGGAAACTCGACT	AAGGTGGTAAAACAAGGACGG
FN1	CGGTGGCTGTCAGTCAAAG	AAACCTCGGCTTCCTCCATAA
UBD	CCGTTCCGAGGAATGGGATTT	GCCATAAGATGAGAGGCTTCTCC
RUNX1	CTGCCCATCGCTTTCAAGGT	GCCGAGTAGTTTTCATCATTGCC
PIK3R2	AAAGGCGGGAACAATAAGCTG	CAACGGAGCAGAAGGTGAGTG
TNF	CCTCTCTCTAATCAGCCCTCTG	GAGGACCTGGGAGTAGATGAG

### Molecular docking studies

The Surflex-Docking docking studies for the designed molecules were performed using module SYBYL-X 2.0 perpetual software. Using ChemDraw Tools, the molecules were sketched and imported and saved into sdf format using open free software from Babel. The co-crystallised protein structures of CEBPD, TP73, ESR2, TAB1 and MAP 3K5 of its PDB code 3L4W, 2XWC, 1U3Q, 5NZZ & 2CLQwas extracted from Protein Data Bank [32–36]. Together with the TRIPOS force field, GasteigerHuckel (GH) charges were added to all designed derivatives for the structure optimization process. Furthermore, energy minimization was carried out using MMFF94s and MMFF94 algorithm process. The processing of protein was accomplished after the incorporation of protein. The co-crystallized ligand and all water molecules were expelled from the crystal structure; more hydrogen was added and the side chain was optimized. TRIPOS force field was used to minimize complexity of structure. The interaction efficiency of the compounds with the receptor was expressed in kcal / mol units by the Surflex-Dock score. The best spot between the protein and the ligand was inserted into the molecular region. The visualization of ligand interaction with receptor is done by using discovery studio visualizer.

## Results

### Identification of DEGs

**Fig. 1:**
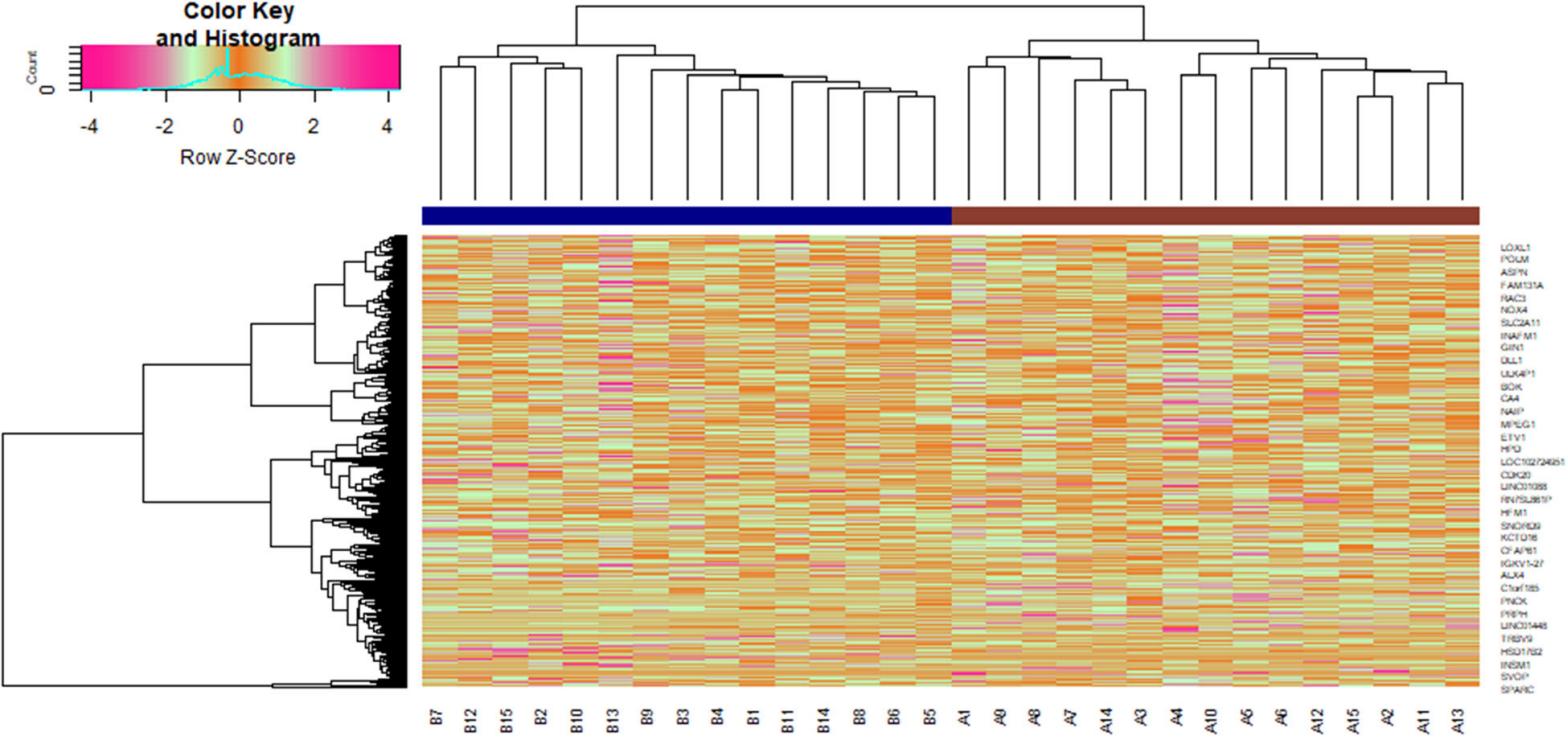
Heat map of differentially expressed genes. Legend on the top left indicate log fold change of genes. (A1 – A15 = metabolically healthy obese samples; B1 – B15 = metabolically unhealthy obese samples)

**Fig. 2:**
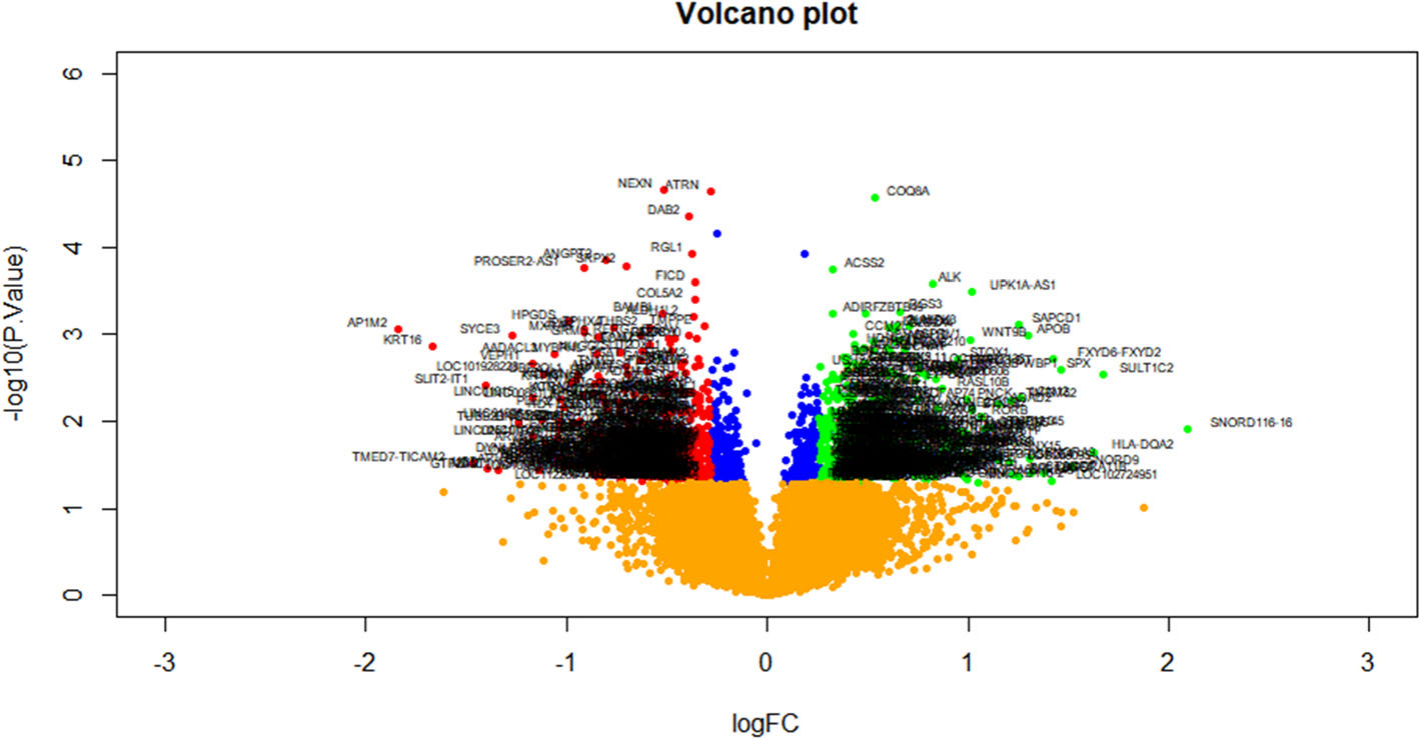
Volcano plot of differentially expressed genes. Genes with a significant change of more than two-fold were selected. Green dot represented up regulated significant genes and red dot represented down regulated significant genes

As presented in the cluster heat map of Fig. 1, total 820 DEGs, comprising 409 up regulated and 411 down regulated genes, were identified between metabolically healthy obese samples and metabolically unhealthy obese samples. DEGs were illustrated by volcano plot (Fig.2), and the top up regulated and down regulated DEGs are listed in Table 2.

**Table 2:** The statistical metrics for key differentially expressed genes (DEGs)

Gene Symbol	logFC	pValue	adj. P.Val	tvalue	Regulation	Gene Name
SNORD116-16	2.091053	0.012212	0.012212	2.677149	Up	small nucleolar RNA, C/D box 116-16
SULT1C2	1.67612	0.002928	0.002928	3.255886	Up	sulfotransferase family 1C member 2
HLA-DQA1	1.62492	0.022453	0.022453	2.41454	Up	major histocompatibility complex, class II, DQ alpha 1
SNORD9	1.51914	0.032752	0.032752	2.245	Up	small nucleolar RNA, C/D box 9
SPX	1.459547	0.00259	0.00259	3.303822	Up	spexin hormone
FXYD6-FXYD2	1.421933	0.001908	0.001908	3.42214	Up	FXYD6-FXYD2 readthrough
LOC102724951	1.41856	0.047463	0.047463	2.072149	Up	uncharacterized LOC102724951
SNORA11B	1.378113	0.039356	0.039356	2.160257	Up	small nucleolar RNA, H/ACA box 11B
CTAGE4	1.31302	0.038788	0.038788	2.16703	Up	CTAGE family member 4
SNORA9	1.308653	0.027236	0.027236	2.328547	Up	small nucleolar RNA, H/ACA box 9
APOB	1.29598	0.001043	0.001043	3.652911	Up	apolipoprotein B
LCN12	1.258207	0.005394	0.005394	3.01344	Up	lipocalin 12
MIR648	1.254627	0.042035	0.042035	2.129481	Up	microRNA 648
SAPCD1	1.251793	0.000762	0.000762	3.771187	Up	suppressor APC domain containing 1
RPL13AP5	1.217507	0.040849	0.040849	2.142888	Up	ribosomal protein L13a pseudogene 5
TMEM52	1.213987	0.005725	0.005725	2.989444	Up	transmembrane protein 52
LOC285095	1.202993	0.029569	0.029569	2.291487	Up	uncharacterized LOC285095
PRICKLE4	1.154227	0.028453	0.028453	2.308873	Up	prickle planar cell polarity protein 4
ADAD2	1.153307	0.006289	0.006289	2.951413	Up	adenosine deaminase domain containing 2
TMEM145	1.111487	0.011906	0.011906	2.687862	Up	transmembrane protein 145
C1orf185	1.084533	0.01231	0.01231	2.673805	Up	chromosome 1 open reading frame 185
RORB	1.0637	0.008779	0.008779	2.814926	Up	RAR related orphan receptor B
MIA	1.05382	0.049316	0.049316	2.053902	Up	MIA SH3 domain containing
PGAM2	1.04492	0.021559	0.021559	2.432446	Up	phosphoglyceratemutase 2
CECR2	1.02018	0.007304	0.007304	2.890514	Up	CECR2 histone acetyl-lysine reader
BTNL8	1.017967	0.007634	0.007634	2.87239	Up	butyrophilin like 8
UPK1A-AS1	1.01654	0.000323	0.000323	4.090921	Up	UPK1A antisense RNA 1
WNT9B	1.010367	0.001141	0.001141	3.618927	Up	Wnt family member 9B
ARL2-SNX15	0.99786	0.023389	0.023389	2.396471	Up	ARL2-SNX15 readthrough (NMD candidate)
PNCK	0.997553	0.005518	0.005518	3.004258	Up	pregnancy up-regulated nonubiquitousCaM kinase
DNM1P46	0.993367	0.014022	0.014022	2.618583	Up	dynamin 1 pseudogene 46
SNORD116-2	0.993207	0.046271	0.046271	2.084217	Up	small nucleolar RNA, C/D box 116-2
SNORA65	0.979973	0.041933	0.041933	2.13062	Up	small nucleolar RNA, H/ACA box 65
NRXN1	0.9751	0.018721	0.018721	2.494208	Up	neurexin 1
LINC01611	0.971233	0.014969	0.014969	2.590702	Up	long intergenic non-protein coding RNA 1611
CFL1P1	0.965867	0.037627	0.037627	2.181137	Up	cofilin 1 pseudogene 1
FAM27C	0.964833	0.013233	0.013233	2.643197	Up	family with sequence similarity 27 member C
TPSD1	0.9607	0.026678	0.026678	2.337832	Up	tryptase delta 1
STOX1	0.952087	0.00188	0.00188	3.427882	Up	storkhead box 1
ARF4-AS1	0.948007	0.019319	0.019319	2.480515	Up	ARF4 antisense RNA 1
PRPH	0.92522	0.002347	0.002347	3.342103	Up	peripherin
FBXO10	0.923973	0.020523	0.020523	2.454076	Up	F-box protein 10
PGM5P3-AS1	0.923733	0.027972	0.027972	2.316545	Up	PGM5P3 antisense RNA 1
LRRC3-DT	0.920987	0.002375	0.002375	3.337439	Up	LRRC3 divergent transcript
INO80B-WBP1	0.919893	0.002585	0.002585	3.304498	Up	INO80B-WBP1 readthrough (NMD candidate)
SORD2P	0.91266	0.018154	0.018154	2.507572	Up	sorbitol dehydrogenase 2, pseudogene
MYLPF	0.896867	0.035595	0.035595	2.206797	Up	myosin light chain, phosphorylatable, fast skeletal muscle
LINC01999	0.894293	0.015657	0.015657	2.571441	Up	long intergenic non-protein coding RNA 1999
CASQ2	0.890407	0.020198	0.020198	2.461069	Up	calsequestrin 2
ULK4P1	0.887613	0.0333	0.0333	2.237399	Up	ULK4 pseudogene 1
PLPP2	0.881447	0.019592	0.019592	2.474383	Up	phospholipid phosphatase 2
CTD-3080P12.3	0.877473	0.011719	0.011719	2.694519	Up	uncharacterized LOC101928857
KIF19	0.875667	0.021475	0.021475	2.434164	Up	kinesin family member 19
KCNK7	0.871247	0.028119	0.028119	2.314193	Up	potassium two pore domain channel subfamily K member 7
RASL10B	0.867707	0.004269	0.004269	3.107011	Up	RAS like family 10 member B
NECAB1	0.860073	0.030586	0.030586	2.276158	Up	N-terminal EF-hand calcium binding protein 1
LINC02055	0.854973	0.030277	0.030277	2.280768	Up	long intergenic non-protein coding RNA 2055
PRODH	0.85302	0.024345	0.024345	2.378692	Up	proline dehydrogenase 1
ZNF236-DT	0.852393	0.012753	0.012753	2.65886	Up	ZNF236 divergent transcript
KCNE2	0.84654	0.007187	0.007187	2.897075	Up	potassium voltage-gated channel subfamily E regulatory subunit 2
AZGP1	0.843307	0.003257	0.003257	3.214022	Up	alpha-2-glycoprotein 1, zinc-binding
CSH1	0.833033	0.044542	0.044542	2.102229	Up	chorionic somatomammotropin hormone 1
ALK	0.82582	0.000259	0.000259	4.172826	Up	ALK receptor tyrosine kinase
CABP1	0.810267	0.030518	0.030518	2.277172	Up	calcium binding protein 1
LOC101927533	0.80322	0.020402	0.020402	2.456678	Up	uncharacterized LOC101927533
SRCIN1	0.794513	0.00297	0.00297	3.250227	Up	SRC kinase signaling inhibitor 1
SOX2-OT	0.791187	0.046179	0.046179	2.085159	Up	SOX2 overlapping transcript
SYCP2L	0.787927	0.042632	0.042632	2.122865	Up	synaptonemal complex protein 2 like
IL17B	0.782547	0.046627	0.046627	2.080586	Up	interleukin 17B
SZT2-AS1	0.777807	0.028194	0.028194	2.312991	Up	SZT2 antisense RNA 1
DMKN	0.77704	0.033651	0.033651	2.232603	Up	dermokine
FASN	0.770993	0.002809	0.002809	3.272056	Up	fatty acid synthase
TAS2R9	0.762667	0.036931	0.036931	2.189782	Up	taste 2 receptor member 9
ANKRD62	0.7576	0.002615	0.002615	3.299996	Up	ankyrin repeat domain 62
LOC101927136	0.755833	0.002251	0.002251	3.358274	Up	uncharacterized LOC101927136
ANKK1	0.753367	0.017117	0.017117	2.533042	Up	ankyrin repeat and kinase domain containing 1
CFAP74	0.743753	0.005581	0.005581	2.999658	Up	cilia and flagella associated protein 74
LYPD6	0.737533	0.00338	0.00338	3.199503	Up	LY6/PLAUR domain containing 6
NAPA-AS1	0.735907	0.034244	0.034244	2.224594	Up	NAPA antisense RNA 1
VWA3A	0.734973	0.041787	0.041787	2.132259	Up	von Willebrand factor A domain containing 3A
PTPRQ	0.73148	0.016897	0.016897	2.538641	Up	protein tyrosine phosphatase receptor type Q
MYOC	0.728007	0.016763	0.016763	2.542067	Up	myocilin
ITIH1	0.724793	0.032609	0.032609	2.246995	Up	inter-alpha-trypsin inhibitor heavy chain 1
HS3ST3A1	0.717073	0.025104	0.025104	2.365016	Up	heparansulfate-glucosamine 3-sulfotransferase 3A1
LINC01933	0.716467	0.011121	0.011121	2.716498	Up	long intergenic non-protein coding RNA 1933
CISH	0.70934	0.011846	0.011846	2.68998	Up	cytokine inducible SH2 containing protein
PVALEF	0.708793	0.011966	0.011966	2.685743	Up	parvalbumin like EF-hand containing
IL12A	0.7084	0.016266	0.016266	2.55505	Up	interleukin 12A
ALPK3	0.707707	0.000804	0.000804	3.751428	Up	alpha kinase 3
DLX1	0.704273	0.011251	0.011251	2.711629	Up	distal-less homeobox 1
LOC105378909	0.692133	0.019505	0.019505	2.476342	Up	uncharacterized LOC105378909
APOM	0.688733	0.02823	0.02823	2.312422	Up	apolipoprotein M
PLIN5	0.68474	0.001495	0.001495	3.5159	Up	perilipin 5
ALDH1L1-AS2	0.6837	0.021431	0.021431	2.435081	Up	ALDH1L1 antisense RNA 2
LOC106660606	0.68294	0.003183	0.003183	3.223079	Up	uncharacterized LOC106660606
NRL	0.672993	0.030217	0.030217	2.281668	Up	neural retina leucine zipper
METTL14-DT	0.669567	0.029186	0.029186	2.29738	Up	METTL14 divergent transcript
ASPRV1	0.660407	0.001149	0.001149	3.616304	Up	aspartic peptidase retroviral like 1
LDB3	0.6577	0.009897	0.009897	2.765213	Up	LIM domain binding 3
LINC02009	0.6576	0.008352	0.008352	2.835496	Up	long intergenic non-protein coding RNA 2009
RGS3	0.657253	0.00054	0.00054	3.900444	Up	regulator of G protein signaling 3
LOC100507144	0.656393	0.031232	0.031232	2.26667	Up	uncharacterized LOC100507144
ANKRD23	0.6563	0.021153	0.021153	2.440814	Up	ankyrin repeat domain 23
NAT14	0.656247	0.012995	0.012995	2.650898	Up	N-acetyltransferase 14 (putative)
RAB26	0.650487	0.049246	0.049246	2.054576	Up	RAB26, member RAS oncogene family
GLUL	0.645487	0.000796	0.000796	3.75498	Up	glutamate-ammonia ligase
REEP2	0.6433	0.021322	0.021322	2.437319	Up	receptor accessory protein 2
CACNB2	0.64148	0.022365	0.022365	2.416279	Up	calcium voltage-gated channel auxiliary subunit beta 2
SLC2A4	0.64038	0.000861	0.000861	3.725632	Up	solute carrier family 2 member 4
TMEM225B	0.639827	0.039209	0.039209	2.162007	Up	transmembrane protein 225B
ADH1B	0.63968	0.001225	0.001225	3.592033	Up	alcohol dehydrogenase 1B (class I), beta polypeptide
OR2A1	0.638853	0.034654	0.034654	2.219125	Up	olfactory receptor family 2 subfamily A member 1
FAM166B	0.63752	0.040943	0.040943	2.141815	Up	family with sequence similarity 166 member B
CPAMD8	0.636207	0.002923	0.002923	3.256463	Up	C3 and PZP like alpha-2-macroglobulin domain containing 8
TMEM63C	0.63106	0.017919	0.017919	2.513237	Up	transmembrane protein 63C
RPS10-NUDT3	0.630793	0.033586	0.033586	2.233482	Up	RPS10-NUDT3 readthrough
GCK	0.630713	0.028465	0.028465	2.308684	Up	glucokinase
PI4KAP1	0.62384	0.017826	0.017826	2.515496	Up	phosphatidylinositol 4-kinase alpha pseudogene 1
TMEM31	0.62244	0.043782	0.043782	2.110345	Up	transmembrane protein 31
CCDC116	0.617427	0.030932	0.030932	2.271057	Up	coiled-coil domain containing 116
NIBAN3	0.616653	0.043308	0.043308	2.115469	Up	niban apoptosis regulator 3
CCNA1	0.61624	0.001648	0.001648	3.478545	Up	cyclin A1
FIRRE	0.611353	0.002658	0.002658	3.293698	Up	firreintergenic repeating RNA element
IZUMO4	0.60368	0.000822	0.000822	3.74308	Up	IZUMO family member 4
CLDN9	0.60176	0.002927	0.002927	3.255992	Up	claudin 9
LINC02210	0.601	0.001418	0.001418	3.536152	Up	long intergenic non-protein coding RNA 2210
CRHR1	0.6002	0.033417	0.033417	2.235799	Up	corticotropin releasing hormone receptor 1
FBXO16	0.599627	0.03972	0.03972	2.155977	Up	F-box protein 16
SHANK2	0.59854	0.014388	0.014388	2.607602	Up	SH3 and multiple ankyrin repeat domains 2
SLIT1	0.596513	0.004491	0.004491	3.086835	Up	slit guidance ligand 1
GLIS1	0.596373	0.043206	0.043206	2.11658	Up	GLIS family zinc finger 1
ACPP	0.594933	0.00541	0.00541	3.01225	Up	acid phosphatase, prostate
CKB	0.590053	0.004051	0.004051	3.127858	Up	creatine kinase B
LINC02242	0.590047	0.011869	0.011869	2.689169	Up	long intergenic non-protein coding RNA 2242
SLC25A52	0.585753	0.016106	0.016106	2.559307	Up	solute carrier family 25 member 52
NR1I3	0.585567	0.008939	0.008939	2.807457	Up	nuclear receptor subfamily 1 group I member 3
KCTD8	0.583867	0.010649	0.010649	2.734665	Up	potassium channel tetramerization domain containing 8
CABP5	0.579207	0.016348	0.016348	2.552868	Up	calcium binding protein 5
POMC	0.57794	0.043371	0.043371	2.114786	Up	proopiomelanocortin
TMEM97	0.57492	0.002776	0.002776	3.276621	Up	transmembrane protein 97
AATK	0.571247	0.027549	0.027549	2.323405	Up	apoptosis associated tyrosine kinase
SLC16A13	0.57088	0.031188	0.031188	2.26731	Up	solute carrier family 16 member 13
BCDIN3D-AS1	0.569787	0.039585	0.039585	2.157557	Up	BCDIN3D antisense RNA 1
CACNA1A	0.569727	0.016613	0.016613	2.545947	Up	calcium voltage-gated channel subunit alpha1 A
TCF15	0.56448	0.005889	0.005889	2.977994	Up	transcription factor 15
CAPN11	0.55676	0.005395	0.005395	3.013368	Up	calpain 11
ARHGEF26	0.556267	0.015335	0.015335	2.580344	Up	Rho guanine nucleotide exchange factor 26
LINC01448	0.550493	0.036509	0.036509	2.19509	Up	long intergenic non-protein coding RNA 1448
CEMP1	0.550073	0.037746	0.037746	2.179666	Up	cementum protein 1
SLC4A4	0.54866	0.011569	0.011569	2.699917	Up	solute carrier family 4 member 4
SLC26A7	0.545633	0.033131	0.033131	2.239734	Up	solute carrier family 26 member 7
MAMDC4	0.54504	0.015232	0.015232	2.583254	Up	MAM domain containing 4
CCNJL	0.541647	0.039394	0.039394	2.159812	Up	cyclin J like
ADGRB3	0.53436	0.012864	0.012864	2.655173	Up	adhesion G protein-coupled receptor B3
COQ8A	0.533247	2.73E-05	2.73E-05	4.994712	Up	coenzyme Q8A
MYRF	0.53202	0.021212	0.021212	2.439579	Up	myelin regulatory factor
GSN-AS1	0.53078	0.001542	0.001542	3.503971	Up	GSN antisense RNA 1
SCN4A	0.52702	0.001219	0.001219	3.593912	Up	sodium voltage-gated channel alpha subunit 4
TBILA	0.521813	0.027316	0.027316	2.327219	Up	TGF-beta induced lncRNA
KCNE5	0.520333	0.029645	0.029645	2.290327	Up	potassium voltage-gated channel subfamily E regulatory subunit 5
KIF26A	0.5177	0.005581	0.005581	2.999708	Up	kinesin family member 26A
DNAJB13	0.517613	0.022862	0.022862	2.406568	Up	DnaJ heat shock protein family (Hsp40) member B13
DDR1	0.517193	0.011322	0.011322	2.708988	Up	discoidin domain receptor tyrosine kinase 1
MCM8-AS1	0.514307	0.033649	0.033649	2.232629	Up	MCM8 antisense RNA 1
ZNF628	0.5143	0.045262	0.045262	2.09466	Up	zinc finger protein 628
WDR62	0.514007	0.011393	0.011393	2.706387	Up	WD repeat domain 62
LINC01252	0.513573	0.040364	0.040364	2.148465	Up	long intergenic non-protein coding RNA 1252
WASHC1	0.50996	0.013544	0.013544	2.633328	Up	WASH complex subunit 1
LOC101927322	0.509507	0.034309	0.034309	2.223715	Up	uncharacterized LOC101927322
SRGAP2-AS1	0.50946	0.0068	0.0068	2.919675	Up	SRGAP2 antisense RNA 1
GCAT	0.50868	0.01609	0.01609	2.559717	Up	glycine C-acetyltransferase
LOC107984875	0.506533	0.00826	0.00826	2.840032	Up	uncharacterized LOC107984875
LOC102723701	0.50454	0.013258	0.013258	2.6424	Up	uncharacterized LOC102723701
FAM53A	0.503847	0.037478	0.037478	2.18297	Up	family with sequence similarity 53 member A
CDKN2C	0.503407	0.039835	0.039835	2.154623	Up	cyclin dependent kinase inhibitor 2C
LRRC56	0.50216	0.027171	0.027171	2.329615	Up	leucine rich repeat containing 56
ZBTB49	0.4927	0.00058	0.00058	3.873677	Up	zinc finger and BTB domain containing 49
SLC19A3	0.491447	0.006315	0.006315	2.949714	Up	solute carrier family 19 member 3
SLED1	0.4908	0.030831	0.030831	2.272538	Up	proteoglycan 3, pro eosinophil major basic protein 2 pseudogene
LINC01303	0.489687	0.049719	0.049719	2.05001	Up	long intergenic non-protein coding RNA 1303
YJEFN3	0.48944	0.048569	0.048569	2.061184	Up	YjeF N-terminal domain containing 3
SNTG2	0.48878	0.013005	0.013005	2.650565	Up	syntrophin gamma 2
TMC2	0.488247	0.03642	0.03642	2.196216	Up	transmembrane channel like 2
ESR2	0.487373	0.006741	0.006741	2.923214	Up	estrogen receptor 2
MOCS1	0.486813	0.002197	0.002197	3.367734	Up	molybdenum cofactor synthesis 1
RARRES1	0.486287	0.036546	0.036546	2.194625	Up	retinoic acid receptor responder 1
SHROOM2	0.48562	0.018206	0.018206	2.506338	Up	shroom family member 2
ISLR2	0.4846	0.025779	0.025779	2.353171	Up	immunoglobulin superfamily containing leucine rich repeat 2
LDHD	0.484293	0.001457	0.001457	3.525893	Up	lactate dehydrogenase D
NEK8	0.480767	0.012581	0.012581	2.664576	Up	NIMA related kinase 8
CASKIN2	0.479527	0.002163	0.002163	3.373832	Up	CASK interacting protein 2
DNAJC27-AS1	0.479133	0.022868	0.022868	2.406453	Up	DNAJC27 antisense RNA 1
RRAD	0.476433	0.045177	0.045177	2.095545	Up	RRAD, Ras related glycolysis inhibitor and calcium channel regulator
CYP2E1	0.47128	0.032339	0.032339	2.250786	Up	cytochrome P450 family 2 subfamily E member 1
TEAD4	0.468247	0.017673	0.017673	2.519224	Up	TEA domain transcription factor 4
DDX11	0.46768	0.048722	0.048722	2.059678	Up	DEAD/H-box helicase 11
NACA4P	0.465847	0.043011	0.043011	2.118704	Up	NACA family member 4, pseudogene
CLDN5	0.46164	0.026624	0.026624	2.338742	Up	claudin 5
GNG7	0.45886	0.014547	0.014547	2.602925	Up	G protein subunit gamma 7
KRT72	0.456787	0.041547	0.041547	2.134956	Up	keratin 72
LINC02809	0.456087	0.006938	0.006938	2.911505	Up	long intergenic non-protein coding RNA 2809
LHFPL5	0.45548	0.020195	0.020195	2.461145	Up	LHFPL tetraspan subfamily member 5
SPTBN4	0.452893	0.002413	0.002413	3.331295	Up	spectrin beta, non-erythrocytic 4
C2CD4C	0.4512	0.034423	0.034423	2.222197	Up	C2 calcium dependent domain containing 4C
ADCK5	0.45064	0.001549	0.001549	3.502228	Up	aarF domain containing kinase 5
DAPK2	0.44706	0.009496	0.009496	2.782402	Up	death associated protein kinase 2
ZNF497	0.44476	0.045315	0.045315	2.094105	Up	zinc finger protein 497
ACVR1C	0.44444	0.003862	0.003862	3.146791	Up	activin A receptor type 1C
FOXI2	0.443987	0.032759	0.032759	2.2449	Up	forkhead box I2
C20orf96	0.443067	0.041914	0.041914	2.130833	Up	chromosome 20 open reading frame 96
SLC9A3R2	0.442747	0.029863	0.029863	2.287008	Up	SLC9A3 regulator 2
KCTD16	0.438287	0.043244	0.043244	2.116163	Up	potassium channel tetramerization domain containing 16
PTGDR	0.436873	0.015729	0.015729	2.569476	Up	prostaglandin D2 receptor
SLC6A8	0.436447	0.002181	0.002181	3.37049	Up	solute carrier family 6 member 8
C16orf95	0.435373	0.017815	0.017815	2.515764	Up	chromosome 16 open reading frame 95
TGM2	0.43472	0.007717	0.007717	2.867995	Up	transglutaminase 2
PFKFB3	0.433267	0.010683	0.010683	2.733323	Up	6-phosphofructo-2-kinase/fructose-2,6-biphosphatase 3
FUT1	0.433133	0.009797	0.009797	2.769451	Up	fucosyltransferase 1 (H blood group)
HDHD3	0.43204	0.001325	0.001325	3.561998	Up	haloaciddehalogenase like hydrolase domain containing 3
SREBF1	0.4311	0.031848	0.031848	2.25777	Up	sterol regulatory element binding transcription factor 1
MAP 2K4P1	0.431067	0.045905	0.045905	2.087978	Up	mitogen-activated protein kinase kinase 4 pseudogene 1
SLC25A21-AS1	0.428013	0.013573	0.013573	2.632439	Up	SLC25A21 antisense RNA 1
GCHFR	0.42692	0.035352	0.035352	2.209947	Up	GTP cyclohydrolase I feedback regulator
MGARP	0.425933	0.017794	0.017794	2.516254	Up	mitochondria localized glutamic acid rich protein
DLL1	0.425747	0.005394	0.005394	3.013381	Up	delta like canonical Notch ligand 1
CCM2L	0.424847	0.000965	0.000965	3.682582	Up	CCM2 like scaffold protein
NPC1L1	0.423333	0.027621	0.027621	2.322233	Up	NPC1 like intracellular cholesterol transporter 1
SLC9A3R1	0.421333	0.011205	0.011205	2.713367	Up	SLC9A3 regulator 1
ALDH1L1	0.419093	0.012238	0.012238	2.676261	Up	aldehyde dehydrogenase 1 family member L1
SMIM10L2B	0.41812	0.028024	0.028024	2.315719	Up	small integral membrane protein 10 like 2B
PLEKHG6	0.414233	0.027165	0.027165	2.329722	Up	pleckstrin homology and RhoGEF domain containing G6
QRICH2	0.413027	0.034715	0.034715	2.218311	Up	glutamine rich 2
DCXR	0.412747	0.007752	0.007752	2.866098	Up	dicarbonyl and L-xylulosereductase
PKN3	0.41206	0.015873	0.015873	2.565566	Up	protein kinase N3
LOC101929457	0.41076	0.010775	0.010775	2.729763	Up	uncharacterized LOC101929457
MAP 3K5	0.410187	0.005026	0.005026	3.0418	Up	mitogen-activated protein kinase kinasekinase 5
SHANK3	0.408153	0.029407	0.029407	2.293968	Up	SH3 and multiple ankyrin repeat domains 3
FGFRL1	0.407573	0.00635	0.00635	2.947475	Up	fibroblast growth factor receptor like 1
CROCCP2	0.406627	0.023876	0.023876	2.387337	Up	CROCC pseudogene 2
LINC01840	0.405807	0.031576	0.031576	2.261679	Up	long intergenic non-protein coding RNA 1840
SLFN13	0.405167	0.010758	0.010758	2.73039	Up	schlafen family member 13
SLC30A3	0.40356	0.022155	0.022155	2.420441	Up	solute carrier family 30 member 3
DOT1L	0.40182	0.002548	0.002548	3.310061	Up	DOT1 like histone lysine methyltransferase
PWWP2B	0.40154	0.012178	0.012178	2.678326	Up	PWWP domain containing 2B
PACSIN1	0.400867	0.013389	0.013389	2.63823	Up	protein kinase C and casein kinase substrate in neurons 1
L3MBTL4	0.40056	0.013628	0.013628	2.630709	Up	L3MBTL histone methyl-lysine binding protein 4
GALNT18	0.399947	0.03634	0.03634	2.197232	Up	polypeptide N-acetylgalactosaminyltransferase 18
SCRN2	0.398233	0.005508	0.005508	3.005004	Up	secernin 2
LIN7A	0.396253	0.001821	0.001821	3.440238	Up	lin-7 homolog A, crumbs cell polarity complex component
TP73	0.396	0.010006	0.010006	2.760666	Up	tumor protein p73
FAHD2B	0.395653	0.003867	0.003867	3.14628	Up	fumarylacetoacetate hydrolase domain containing 2B
LINC01550	0.395573	0.02393	0.02393	2.386337	Up	long intergenic non-protein coding RNA 1550
GS1-124K5.11	0.39448	0.002126	0.002126	3.380439	Up	RAB guanine nucleotide exchange factor 1 pseudogene
KCTD17	0.39394	0.039791	0.039791	2.155139	Up	potassium channel tetramerization domain containing 17
KLF15	0.393687	0.011645	0.011645	2.697181	Up	Kruppel like factor 15
ATP2C2-AS1	0.391593	0.028443	0.028443	2.309025	Up	ATP2C2 antisense RNA 1
SYT7	0.391587	0.043499	0.043499	2.113395	Up	synaptotagmin 7
IRAK2	0.390753	0.038967	0.038967	2.164885	Up	interleukin 1 receptor associated kinase 2
HELZ2	0.388267	0.036382	0.036382	2.196698	Up	helicase with zinc finger 2
MLXIPL	0.386427	0.010972	0.010972	2.722173	Up	MLX interacting protein like
GINS3	0.386213	0.018589	0.018589	2.4973	Up	GINS complex subunit 3
EGFLAM	0.385367	0.018454	0.018454	2.500454	Up	EGF like, fibronectin type III and laminin G domains
CASC16	0.384113	0.03905	0.03905	2.163893	Up	cancer susceptibility 16
BOK	0.382033	0.00183	0.00183	3.438406	Up	BCL2 family apoptosis regulator BOK
ADCY4	0.380647	0.007812	0.007812	2.862943	Up	adenylatecyclase 4
SPHK1	0.378633	0.043493	0.043493	2.113465	Up	sphingosine kinase 1
PPP1R16A	0.378367	0.041909	0.041909	2.130896	Up	protein phosphatase 1 regulatory subunit 16A
HOMEZ	0.3768	0.003024	0.003024	3.243199	Up	homeobox and leucine zipper encoding
TAF1C	0.375107	0.020069	0.020069	2.463884	Up	TATA-box binding protein associated factor, RNA polymerase I subunit C
PDCD2L	0.373413	0.005258	0.005258	3.023712	Up	programmed cell death 2 like
DEPP1	0.36932	0.043836	0.043836	2.109767	Up	DEPP1 autophagy regulator
CDK20	0.369047	0.040925	0.040925	2.142014	Up	cyclin dependent kinase 20
KIFC2	0.36856	0.011059	0.011059	2.718839	Up	kinesin family member C2
LOC101927495	0.36686	0.042416	0.042416	2.125249	Up	uncharacterized LOC101927495
MED16	0.3667	0.031179	0.031179	2.26744	Up	mediator complex subunit 16
PRCD	0.365607	0.008088	0.008088	2.848706	Up	photoreceptor disc component
CD320	0.365453	0.014275	0.014275	2.610964	Up	CD320 molecule
LLGL2	0.365393	0.034201	0.034201	2.225165	Up	LLGL scribble cell polarity complex component 2
ECHDC3	0.36472	0.023262	0.023262	2.398894	Up	enoyl-CoA hydratase domain containing 3
GK	0.364333	0.039685	0.039685	2.156382	Up	glycerol kinase
MARCHF3	0.361713	0.022439	0.022439	2.414818	Up	membrane associated ring-CH-type finger 3
GRASP	0.359927	0.014748	0.014748	2.597057	Up	general receptor for phosphoinositides 1 associated scaffold protein
NBPF10	0.359027	0.017461	0.017461	2.524435	Up	NBPF member 10
SLC25A22	0.358167	0.028361	0.028361	2.310329	Up	solute carrier family 25 member 22
FRMD1	0.355467	0.014648	0.014648	2.599955	Up	FERM domain containing 1
GNG3	0.35502	0.041873	0.041873	2.131292	Up	G protein subunit gamma 3
PTPN3	0.35484	0.017609	0.017609	2.520785	Up	protein tyrosine phosphatase non-receptor type 3
ZNF775	0.354047	0.010894	0.010894	2.725137	Up	zinc finger protein 775
CEBPD	0.35346	0.032804	0.032804	2.244272	Up	CCAAT enhancer binding protein delta
EDA	0.353147	0.026661	0.026661	2.338123	Up	ectodysplasin A
CPLX1	0.353027	0.046099	0.046099	2.085981	Up	complexin 1
SLC35G2	0.352027	0.003293	0.003293	3.209745	Up	solute carrier family 35 member G2
LGALS12	0.351687	0.025859	0.025859	2.351799	Up	galectin 12
CNTFR	0.350407	0.039045	0.039045	2.163954	Up	ciliaryneurotrophic factor receptor
KANK3	0.349893	0.030518	0.030518	2.277178	Up	KN motif and ankyrin repeat domains 3
TPRN	0.349767	0.01286	0.01286	2.655322	Up	taperin
INAFM1	0.349327	0.033182	0.033182	2.239034	Up	InaF motif containing 1
SLC2A11	0.349233	0.003274	0.003274	3.212037	Up	solute carrier family 2 member 11
ZNF784	0.349	0.01501	0.01501	2.589523	Up	zinc finger protein 784
MKNK2	0.348927	0.004343	0.004343	3.100189	Up	MAPK interacting serine/threonine kinase 2
UBTD1	0.348413	0.022659	0.022659	2.4105	Up	ubiquitin domain containing 1
NBR2	0.348333	0.035792	0.035792	2.204241	Up	neighbor of BRCA1 lncRNA 2
POLM	0.34684	0.013365	0.013365	2.638976	Up	DNA polymerase mu
GPIHBP1	0.346247	0.025151	0.025151	2.364191	Up	glycosylphosphatidylinositol anchored high density lipoprotein binding protein 1
GLYCTK	0.345573	0.037605	0.037605	2.181401	Up	glycerate kinase
LINC01179	0.345427	0.007842	0.007842	2.861379	Up	long intergenic non-protein coding RNA 1179
GPT2	0.345107	0.005711	0.005711	2.990416	Up	glutamic--pyruvic transaminase 2
RABL2B	0.344613	0.008317	0.008317	2.837186	Up	RAB, member of RAS oncogene family like 2B
MIB2	0.343647	0.016939	0.016939	2.537552	Up	mindbomb E3 ubiquitin protein ligase 2
TNFRSF8	0.342827	0.028406	0.028406	2.309619	Up	TNF receptor superfamily member 8
EPB41L4B	0.341027	0.049728	0.049728	2.049921	Up	erythrocyte membrane protein band 4.1 like 4B
CENPX	0.34044	0.004819	0.004819	3.058676	Up	centromere protein X
PXMP2	0.338413	0.021778	0.021778	2.427992	Up	peroxisomal membrane protein 2
CA4	0.33818	0.025048	0.025048	2.366019	Up	carbonic anhydrase 4
RDH13	0.337287	0.016751	0.016751	2.54239	Up	retinol dehydrogenase 13
WNT11	0.337087	0.024224	0.024224	2.380901	Up	Wnt family member 11
ZNF727	0.336547	0.02561	0.02561	2.356123	Up	zinc finger protein 727
TMEM143	0.335967	0.032586	0.032586	2.247312	Up	transmembrane protein 143
WDR90	0.333773	0.023394	0.023394	2.39637	Up	WD repeat domain 90
CARD10	0.331487	0.026367	0.026367	2.343088	Up	caspase recruitment domain family member 10
CCDC88C	0.329653	0.00813	0.00813	2.846562	Up	coiled-coil domain containing 88C
GPHN	0.32896	0.003334	0.003334	3.204898	Up	gephyrin
CASP16P	0.328467	0.039239	0.039239	2.161647	Up	caspase 16, pseudogene
ADIRF	0.32752	0.000578	0.000578	3.874897	Up	adipogenesis regulatory factor
PCDHGC4	0.32742	0.037028	0.037028	2.188563	Up	protocadherin gamma subfamily C, 4
KIAA0895L	0.327093	0.005065	0.005065	3.038666	Up	KIAA0895 like
ACSS2	0.325927	0.000183	0.000183	4.301369	Up	acyl-CoA synthetase short chain family member 2
LOC100996842	0.325027	0.042419	0.042419	2.125216	Up	uncharacterized LOC100996842
ZNF366	0.324253	0.0029	0.0029	3.25961	Up	zinc finger protein 366
EBP	0.321927	0.011617	0.011617	2.698192	Up	EBP cholestenol delta-isomerase
BSN	0.32112	0.02936	0.02936	2.294702	Up	bassoon presynaptic cytomatrix protein
DHRS3	0.319227	0.003256	0.003256	3.214186	Up	dehydrogenase/reductase 3
OPLAH	0.317693	0.008179	0.008179	2.844112	Up	5-oxoprolinase, ATP-hydrolysing
SETD1A	0.3171	0.035833	0.035833	2.203722	Up	SET domain containing 1A, histone lysine methyltransferase
TYSND1	0.31578	0.007605	0.007605	2.873964	Up	trypsin domain containing 1
TRMT1L	0.314633	0.010321	0.010321	2.74775	Up	tRNAmethyltransferase 1 like
EDNRA	0.311733	0.049481	0.049481	2.052308	Up	endothelin receptor type A
TDRKH	0.31074	0.028108	0.028108	2.314361	Up	tudor and KH domain containing
EXD3	0.310607	0.040952	0.040952	2.14171	Up	exonuclease 3'-5' domain containing 3
PEAR1	0.308427	0.037173	0.037173	2.186759	Up	platelet endothelial aggregation receptor 1
LMLN	0.30822	0.010311	0.010311	2.748134	Up	leishmanolysin like peptidase
AASS	0.308027	0.039253	0.039253	2.161477	Up	aminoadipate-semialdehyde synthase
LRRC29	0.308	0.040371	0.040371	2.148389	Up	leucine rich repeat containing 29
EIF1B-AS1	0.307667	0.014918	0.014918	2.592162	Up	EIF1B antisense RNA 1
MPND	0.307367	0.01476	0.01476	2.596721	Up	MPN domain containing
SPHK2	0.30734	0.045158	0.045158	2.095746	Up	sphingosine kinase 2
VPS37B	0.306187	0.009521	0.009521	2.781321	Up	VPS37B subunit of ESCRT-I
ZNF821	0.304933	0.029371	0.029371	2.294531	Up	zinc finger protein 821
EPHB4	0.304347	0.016088	0.016088	2.559768	Up	EPH receptor B4
MICALL1	0.30348	0.029928	0.029928	2.286026	Up	MICAL like 1
CENPS	0.303127	0.00445	0.00445	3.090472	Up	centromere protein S
RAC3	0.302293	0.024619	0.024619	2.373715	Up	Rac family small GTPase 3
SAG	0.302187	0.043818	0.043818	2.109961	Up	S-antigen visual arrestin
ADHFE1	0.301933	0.013006	0.013006	2.650532	Up	alcohol dehydrogenase iron containing 1
LINC01460	0.301607	0.014749	0.014749	2.597019	Up	long intergenic non-protein coding RNA 1460
ACAT2	0.301127	0.049893	0.049893	2.048342	Up	acetyl-CoA acetyltransferase 2
USHBP1	0.300987	0.010881	0.010881	2.725632	Up	USH1 protein network component harmonin binding protein 1
RAPGEF3	0.300947	0.02845	0.02845	2.308918	Up	Rap guanine nucleotide exchange factor 3
EP400P1	0.300847	0.035764	0.035764	2.204603	Up	EP400 pseudogene 1
CSPG4	0.3001	0.005719	0.005719	2.98983	Up	chondroitin sulfate proteoglycan 4
TMEM53	0.29854	0.020161	0.020161	2.461868	Up	transmembrane protein 53
PMM1	0.297573	0.014532	0.014532	2.603367	Up	phosphomannomutase 1
ARHGEF15	0.29746	0.049419	0.049419	2.052904	Up	Rho guanine nucleotide exchange factor 15
CD3EAP	0.296887	0.037923	0.037923	2.177497	Up	CD3e molecule associated protein
IMPA2	0.296727	0.033772	0.033772	2.230953	Up	inositol monophosphatase 2
PUSL1	0.296007	0.036908	0.036908	2.190066	Up	pseudouridine synthase like 1
MRPL11	0.29498	0.003492	0.003492	3.186591	Up	mitochondrial ribosomal protein L11
RRP1	0.294953	0.013034	0.013034	2.649615	Up	ribosomal RNA processing 1
C19orf25	0.294907	0.024903	0.024903	2.368605	Up	chromosome 19 open reading frame 25
LINC01128	0.294653	0.028588	0.028588	2.306742	Up	long intergenic non-protein coding RNA 1128
PSEN2	0.292733	0.016252	0.016252	2.555403	Up	presenilin 2
MVD	0.29174	0.049805	0.049805	2.049181	Up	mevalonatediphosphate decarboxylase
FLJ31104	0.29174	0.041537	0.041537	2.135077	Up	uncharacterized LOC441072
IRX6	0.287387	0.049491	0.049491	2.05221	Up	iroquoishomeobox 6
GGT6	0.285007	0.03879	0.03879	2.167003	Up	gamma-glutamyltransferase 6
HOXA6	0.284887	0.036879	0.036879	2.190424	Up	homeobox A6
SLC25A10	0.28452	0.046013	0.046013	2.086869	Up	solute carrier family 25 member 10
TBC1D7	0.284187	0.014585	0.014585	2.601801	Up	TBC1 domain family member 7
GPR146	0.283147	0.009124	0.009124	2.798966	Up	G protein-coupled receptor 146
LOC648987	0.282687	0.014648	0.014648	2.599972	Up	uncharacterized LOC648987
RASIP1	0.28128	0.025608	0.025608	2.356149	Up	Ras interacting protein 1
THAP3	0.28086	0.005152	0.005152	3.031874	Up	THAP domain containing 3
SH3D21	0.279873	0.047297	0.047297	2.073815	Up	SH3 domain containing 21
ZNF598	0.279487	0.036933	0.036933	2.189749	Up	zinc finger protein 598
VAMP2	0.278927	0.036654	0.036654	2.193255	Up	vesicle associated membrane protein 2
FAM131A	0.277193	0.01078	0.01078	2.729571	Up	family with sequence similarity 131 member A
PLEKHH3	0.276973	0.021042	0.021042	2.443126	Up	pleckstrin homology, MyTH4 and FERM domain containing H3
MPST	0.27696	0.044439	0.044439	2.103324	Up	mercaptopyruvatesulfurtransferase
EGFL7	0.27696	0.043764	0.043764	2.110539	Up	EGF like domain multiple 7
NFKBIL1	0.27696	0.02297	0.02297	2.404471	Up	NFKB inhibitor like 1
STBD1	0.274827	0.021907	0.021907	2.425394	Up	starch binding domain 1
SMIM10L2A	0.274493	0.0499	0.0499	2.048272	Up	small integral membrane protein 10 like 2A
SNHG20	0.274367	0.023088	0.023088	2.402207	Up	small nucleolar RNA host gene 20
ARSG	0.27384	0.042052	0.042052	2.1293	Up	arylsulfatase G
DANCR	0.272867	0.039453	0.039453	2.159115	Up	differentiation antagonizing non-protein coding RNA
TNS2	0.27146	0.021422	0.021422	2.435265	Up	tensin 2
SVOP	0.270813	0.040165	0.040165	2.150772	Up	SV2 related protein
TAB1	0.269447	0.04564	0.04564	2.090719	Up	TGF-beta activated kinase 1 (MAP 3K7) binding protein 1
SEMA4C	0.26936	0.012182	0.012182	2.678211	Up	semaphorin 4C
HADH	0.267253	0.017346	0.017346	2.527301	Up	hydroxyacyl-CoA dehydrogenase
USH2A	0.26662	0.002348	0.002348	3.34197	Up	usherin
ACADS	0.266613	0.026468	0.026468	2.341366	Up	acyl-CoA dehydrogenase short chain
HLA-F	0.266587	0.026586	0.026586	2.339375	Up	major histocompatibility complex, class I, F
MAIP1	0.26484	0.025931	0.025931	2.350542	Up	matrix AAA peptidase interacting protein 1
PCBD2	0.264567	0.010857	0.010857	2.726568	Up	pterin-4 alpha-carbinolaminedehydratase 2
IFI35	0.263513	0.04722	0.04722	2.074586	Up	interferon induced protein 35
POU6F1	0.261913	0.0477	0.0477	2.069781	Up	POU class 6 homeobox 1
GUCD1	0.261527	0.018894	0.018894	2.490219	Up	guanylylcyclase domain containing 1
FAHD2CP	0.260187	0.010239	0.010239	2.751057	Up	fumarylacetoacetate hydrolase domain containing 2C, pseudogene
AP1M2	-1.83963	0.000873	0.000873	-3.7202	Down	adaptor related protein complex 1 subunit mu 2
KRT16	-1.66819	0.001376	0.001376	-3.54761	Down	keratin 16
TMED7-TICAM2	-1.46751	0.03117	0.03117	-2.26757	Down	TMED7-TICAM2 readthrough
SLIT2-IT1	-1.40593	0.003861	0.003861	-3.1469	Down	SLIT2 intronic transcript 1
UBD	-1.39199	0.034472	0.034472	-2.22154	Down	ubiquitin D
GTF2A1L	-1.34071	0.036078	0.036078	-2.20057	Down	general transcription factor IIA subunit 1 like
SYCE3	-1.27163	0.001032	0.001032	-3.65703	Down	synaptonemal complex central element protein 3
TUBB2B	-1.23847	0.010346	0.010346	-2.74672	Down	tubulin beta 2B class IIb
LINC01315	-1.17362	0.005379	0.005379	-3.01454	Down	long intergenic non-protein coding RNA 1315
LINC02541	-1.17343	0.015393	0.015393	-2.57875	Down	long intergenic non-protein coding RNA 2541
VEPH1	-1.17286	0.002124	0.002124	-3.38071	Down	ventricular zone expressed PH domain containing 1
NDST1-AS1	-1.14183	0.036221	0.036221	-2.19875	Down	NDST1 antisense RNA 1
LINC01876	-1.12505	0.009476	0.009476	-2.78327	Down	long intergenic non-protein coding RNA 1876
LOC101928228	-1.11811	0.002831	0.002831	-3.26903	Down	uncharacterized LOC101928228
ARL5C	-1.10639	0.017561	0.017561	-2.52198	Down	ADP ribosylation factor like GTPase 5C
DYNLRB2	-1.08251	0.024003	0.024003	-2.38498	Down	dynein light chain roadblock-type 2
H1-1	-1.06444	0.020425	0.020425	-2.45617	Down	H1.1 linker histone, cluster member
AADACL3	-1.06131	0.001719	0.001719	-3.46237	Down	arylacetamidedeacetylase like 3
NPIPB8	-1.04889	0.026769	0.026769	-2.33631	Down	nuclear pore complex interacting protein family member B8
LINC01909	-1.04454	0.015374	0.015374	-2.57927	Down	long intergenic non-protein coding RNA 1909
CCL18	-1.03957	0.012174	0.012174	-2.67847	Down	C-C motif chemokine ligand 18
SFRP2	-1.03733	0.010127	0.010127	-2.75565	Down	secreted frizzled related protein 2
LINC00861	-1.03042	0.005653	0.005653	-2.9945	Down	long intergenic non-protein coding RNA 861
PLEK2	-1.01229	0.006901	0.006901	-2.91366	Down	pleckstrin 2
CFAP61	-1.01106	0.033284	0.033284	-2.23762	Down	cilia and flagella associated protein 61
HPGDS	-0.9886	0.000699	0.000699	-3.80361	Down	hematopoietic prostaglandin D synthase
KRT14	-0.97832	0.003559	0.003559	-3.17915	Down	keratin 14
SERF1A	-0.97264	0.031251	0.031251	-2.26638	Down	small EDRK-rich factor 1A
LINC02621	-0.96818	0.010579	0.010579	-2.73741	Down	long intergenic non-protein coding RNA 2621
KCNA1	-0.95145	0.003218	0.003218	-3.21883	Down	potassium voltage-gated channel subfamily A member 1
ANKRD1	-0.94966	0.020504	0.020504	-2.45449	Down	ankyrin repeat domain 1
UBE2QL1	-0.94181	0.002868	0.002868	-3.26387	Down	ubiquitin conjugating enzyme E2 Q family like 1
C15orf65	-0.93809	0.010552	0.010552	-2.73851	Down	chromosome 15 open reading frame 65
OLAH	-0.93508	0.010115	0.010115	-2.75615	Down	oleoyl-ACP hydrolase
ARHGEF9-IT1	-0.92943	0.032287	0.032287	-2.25152	Down	ARHGEF9 intronic transcript 1
KCNA2	-0.92134	0.004863	0.004863	-3.05502	Down	potassium voltage-gated channel subfamily A member 2
MXRA5	-0.91562	0.000951	0.000951	-3.68797	Down	matrix remodeling associated 5
JPH2	-0.91528	0.000873	0.000873	-3.72019	Down	junctophilin 2
C17orf99	-0.91307	0.022929	0.022929	-2.40527	Down	chromosome 17 open reading frame 99
PROSER2-AS1	-0.90959	0.000174	0.000174	-4.31999	Down	PROSER2 antisense RNA 1
FYB2	-0.90903	0.022109	0.022109	-2.42135	Down	FYN binding protein 2
PTPRZ1	-0.89926	0.026794	0.026794	-2.33589	Down	protein tyrosine phosphatase receptor type Z1
ITGB1BP2	-0.89303	0.015913	0.015913	-2.56447	Down	integrin subunit beta 1 binding protein 2
ZFHX4-AS1	-0.88985	0.034802	0.034802	-2.21716	Down	ZFHX4 antisense RNA 1
LOC100505622	-0.88851	0.026329	0.026329	-2.34373	Down	uncharacterized LOC100505622
LOC107986163	-0.8756	0.037842	0.037842	-2.17849	Down	uncharacterized LOC107986163
GABRB2	-0.87298	0.014162	0.014162	-2.61436	Down	gamma-aminobutyric acid type A receptor beta2 subunit
CELF4	-0.8686	0.024073	0.024073	-2.38368	Down	CUGBP Elav-like family member 4
C4BPB	-0.86515	0.026739	0.026739	-2.33681	Down	complement component 4 binding protein beta
ATRNL1	-0.86285	0.004747	0.004747	-3.06464	Down	attractin like 1
INHBA	-0.85583	0.003642	0.003642	-3.16997	Down	inhibin subunit beta A
LINC00323	-0.85416	0.021827	0.021827	-2.42701	Down	long intergenic non-protein coding RNA 323
FAM151A	-0.85212	0.042001	0.042001	-2.12987	Down	family with sequence similarity 151 member A
STKLD1	-0.85192	0.01049	0.01049	-2.74094	Down	serine/threonine kinase like domain containing 1
NMRAL2P	-0.85008	0.038159	0.038159	-2.17462	Down	NmrA like redox sensor 2, pseudogene
MYBPHL	-0.84666	0.001678	0.001678	-3.47163	Down	myosin binding protein H like
GRM5	-0.84575	0.001062	0.001062	-3.64624	Down	glutamate metabotropic receptor 5
LBP	-0.84142	0.003065	0.003065	-3.23785	Down	lipopolysaccharide binding protein
SEZ6	-0.83764	0.01322	0.01322	-2.64362	Down	seizure related 6 homolog
CACNG2	-0.82779	0.003396	0.003396	-3.19763	Down	calcium voltage-gated channel auxiliary subunit gamma 2
OLR1	-0.82579	0.044364	0.044364	-2.10412	Down	oxidized low density lipoprotein receptor 1
CHST4	-0.81618	0.005129	0.005129	-3.03367	Down	carbohydrate sulfotransferase 4
SERPINE1	-0.8149	0.023386	0.023386	-2.39654	Down	serpin family E member 1
ACSM1	-0.81072	0.006117	0.006117	-2.96266	Down	acyl-CoA synthetase medium chain family member 1
BPI	-0.80597	0.019588	0.019588	-2.47448	Down	bactericidal permeability increasing protein
ANGPT2	-0.80309	0.000141	0.000141	-4.3973	Down	angiopoietin 2
HLA-DQA1	-0.79472	0.007581	0.007581	-2.87525	Down	major histocompatibility complex, class II, DQ alpha 1
LOC105371050	-0.79405	0.025335	0.025335	-2.36094	Down	uncharacterized LOC105371050
KBTBD12	-0.77889	0.013931	0.013931	-2.62137	Down	kelch repeat and BTB domain containing 12
VSTM2L	-0.77399	0.040074	0.040074	-2.15183	Down	V-set and transmembrane domain containing 2 like
PDPN	-0.77141	0.022771	0.022771	-2.40832	Down	podoplanin
SPESP1	-0.76823	0.018515	0.018515	-2.49903	Down	sperm equatorial segment protein 1
EPHX4	-0.76699	0.000847	0.000847	-3.73178	Down	epoxide hydrolase 4
LINC01366	-0.76575	0.020048	0.020048	-2.46434	Down	long intergenic non-protein coding RNA 1366
RAMP1	-0.75425	0.03836	0.03836	-2.17218	Down	receptor activity modifying protein 1
APLN	-0.75123	0.035932	0.035932	-2.20245	Down	apelin
BCYRN1	-0.75046	0.0142	0.0142	-2.61323	Down	brain cytoplasmic RNA 1
PROM1	-0.74233	0.016077	0.016077	-2.56008	Down	prominin 1
EMBP1	-0.74181	0.006074	0.006074	-2.9655	Down	embiginpseudogene 1
EPHB3	-0.73908	0.006539	0.006539	-2.93556	Down	EPH receptor B3
ASAH2	-0.73624	0.008099	0.008099	-2.84812	Down	N-acylsphingosineamidohydrolase 2
TMLHE-AS1	-0.73541	0.03531	0.03531	-2.21049	Down	TMLHE antisense RNA 1
NUGGC	-0.73261	0.001609	0.001609	-3.4879	Down	nuclear GTPase, germinal center associated
LOC112268408	-0.73205	0.047435	0.047435	-2.07242	Down	uncharacterized LOC112268408
SCOC-AS1	-0.73056	0.029921	0.029921	-2.28613	Down	SCOC antisense RNA 1
CYTL1	-0.72915	0.046418	0.046418	-2.08271	Down	cytokine like 1
LINC01861	-0.72737	0.005976	0.005976	-2.97207	Down	long intergenic non-protein coding RNA 1861
DRD1	-0.71583	0.006026	0.006026	-2.96871	Down	dopamine receptor D1
DDX43	-0.71493	0.029747	0.029747	-2.28877	Down	DEAD-box helicase 43
LINGO1	-0.71183	0.004311	0.004311	-3.10312	Down	leucine rich repeat and Ig domain containing 1
HSD11B1	-0.70994	0.009219	0.009219	-2.7947	Down	hydroxysteroid 11-beta dehydrogenase 1
SRPX2	-0.70059	0.000163	0.000163	-4.34405	Down	sushi repeat containing protein X-linked 2
TNMD	-0.69969	0.002375	0.002375	-3.33749	Down	tenomodulin
CYP2C9	-0.69773	0.038159	0.038159	-2.17462	Down	cytochrome P450 family 2 subfamily C member 9
MFAP5	-0.69703	0.002943	0.002943	-3.25378	Down	microfibril associated protein 5
LINC00449	-0.69067	0.032549	0.032549	-2.24783	Down	long intergenic non-protein coding RNA 449
PAGE4	-0.67675	0.02332	0.02332	-2.39778	Down	PAGE family member 4
LINC02688	-0.67659	0.040433	0.040433	-2.14768	Down	long intergenic non-protein coding RNA 2688
LINC02384	-0.67644	0.01088	0.01088	-2.72568	Down	long intergenic non-protein coding RNA 2384
KRT5	-0.66945	0.020488	0.020488	-2.45483	Down	keratin 5
PLA2G2C	-0.66438	0.021408	0.021408	-2.43554	Down	phospholipase A2 group IIC
SLC47A1	-0.66283	0.004917	0.004917	-3.05058	Down	solute carrier family 47 member 1
KRTAP5-8	-0.66176	0.013759	0.013759	-2.62665	Down	keratin associated protein 5-8
FASLG	-0.65842	0.024609	0.024609	-2.37389	Down	Fas ligand
LINC01088	-0.6579	0.007562	0.007562	-2.87629	Down	long intergenic non-protein coding RNA 1088
HMGA2-AS1	-0.65634	0.027717	0.027717	-2.32068	Down	HMGA2 antisense RNA 1
TNFRSF12A	-0.64562	0.036314	0.036314	-2.19757	Down	TNF receptor superfamily member 12A
CCDC122	-0.64133	0.026547	0.026547	-2.34004	Down	coiled-coil domain containing 122
PSAT1	-0.63545	0.001949	0.001949	-3.41395	Down	phosphoserine aminotransferase 1
F13A1	-0.63391	0.006252	0.006252	-2.95381	Down	coagulation factor XIII A chain
DHH	-0.63179	0.021762	0.021762	-2.42833	Down	desert hedgehog signaling molecule
RERG	-0.63159	0.001024	0.001024	-3.65989	Down	RAS like estrogen regulated growth inhibitor
BLOC1S5-TXNDC5	-0.6295	0.027925	0.027925	-2.3173	Down	BLOC1S5-TXNDC5 readthrough (NMD candidate)
LOC101927708	-0.62838	0.031922	0.031922	-2.25671	Down	uncharacterized LOC101927708
SLC4A3	-0.62674	0.017538	0.017538	-2.52255	Down	solute carrier family 4 member 3
FLNC	-0.62642	0.020204	0.020204	-2.46095	Down	filamin C
AMPD1	-0.62482	0.047649	0.047649	-2.07029	Down	adenosine monophosphate deaminase 1
SLIT2	-0.624	0.001565	0.001565	-3.49831	Down	slit guidance ligand 2
LYVE1	-0.62117	0.010171	0.010171	-2.75382	Down	lymphatic vessel endothelial hyaluronan receptor 1
C16orf71	-0.61966	0.032589	0.032589	-2.24728	Down	chromosome 16 open reading frame 71
HFM1	-0.6184	0.01019	0.01019	-2.75307	Down	helicase for meiosis 1
FRZB	-0.6172	0.043793	0.043793	-2.11023	Down	frizzled related protein
ANKRD44-IT1	-0.61691	0.019758	0.019758	-2.47071	Down	ANKRD44 intronic transcript 1
IQCH	-0.61355	0.024461	0.024461	-2.37658	Down	IQ motif containing H
HSD17B2	-0.61154	0.0392	0.0392	-2.16211	Down	hydroxysteroid 17-beta dehydrogenase 2
HTR7	-0.61001	0.020491	0.020491	-2.45477	Down	5-hydroxytryptamine receptor 7
TIGIT	-0.60797	0.038413	0.038413	-2.17154	Down	T cell immunoreceptor with Ig and ITIM domains
CD28	-0.60719	0.005482	0.005482	-3.00687	Down	CD28 molecule
RFX4	-0.60674	0.023241	0.023241	-2.39928	Down	regulatory factor X4
LRRTM2	-0.60411	0.016618	0.016618	-2.54583	Down	leucine rich repeat transmembrane neuronal 2
THBS1	-0.60387	0.012722	0.012722	-2.65989	Down	thrombospondin 1
LINC01376	-0.6004	0.01737	0.01737	-2.5267	Down	long intergenic non-protein coding RNA 1376
FCGR1B	-0.59985	0.037964	0.037964	-2.177	Down	Fc fragment of IgG receptor Ib
ADAMTS6	-0.59864	0.002691	0.002691	-3.28887	Down	ADAM metallopeptidase with thrombospondin type 1 motif 6
SLC6A1	-0.59683	0.019212	0.019212	-2.48293	Down	solute carrier family 6 member 1
NEK10	-0.59117	0.0202	0.0202	-2.46102	Down	NIMA related kinase 10
SYT12	-0.59044	0.010636	0.010636	-2.73517	Down	synaptotagmin 12
THBS2	-0.58761	0.000849	0.000849	-3.73069	Down	thrombospondin 2
PEMT	-0.58693	0.042542	0.042542	-2.12385	Down	phosphatidylethanolamine N-methyltransferase
CLEC4M	-0.58663	0.001339	0.001339	-3.55795	Down	C-type lectin domain family 4 member M
HTR2B	-0.58465	0.012081	0.012081	-2.68169	Down	5-hydroxytryptamine receptor 2B
DUXAP8	-0.58371	0.037364	0.037364	-2.18438	Down	double homeobox A pseudogene 8
DAO	-0.58274	0.015121	0.015121	-2.58637	Down	D-amino acid oxidase
ZNF208	-0.57918	0.007614	0.007614	-2.87346	Down	zinc finger protein 208
ODF3L1	-0.57736	0.013767	0.013767	-2.62641	Down	outer dense fiber of sperm tails 3 like 1
TFAP2C	-0.57669	0.03794	0.03794	-2.17729	Down	transcription factor AP-2 gamma
TNF	-0.57654	0.037316	0.037316	-2.18498	Down	tumor necrosis factor
GRB14	-0.57595	0.037375	0.037375	-2.18424	Down	growth factor receptor bound protein 14
FAP	-0.57409	0.016017	0.016017	-2.56167	Down	fibroblast activation protein alpha
ZBTB20-AS1	-0.57301	0.034421	0.034421	-2.22222	Down	ZBTB20 antisense RNA 1
TRIM63	-0.57147	0.026027	0.026027	-2.3489	Down	tripartite motif containing 63
KCNG2	-0.57145	0.033467	0.033467	-2.23511	Down	potassium voltage-gated channel modifier subfamily G member 2
LOC101929130	-0.57061	0.024183	0.024183	-2.38166	Down	uncharacterized LOC101929130
CCDC136	-0.56956	0.01757	0.01757	-2.52176	Down	coiled-coil domain containing 136
LYPLAL1-DT	-0.56901	0.033136	0.033136	-2.23967	Down	LYPLAL1 divergent transcript
SULT1A1	-0.56533	0.015341	0.015341	-2.58019	Down	sulfotransferase family 1A member 1
LINC02728	-0.56482	0.041126	0.041126	-2.13972	Down	long intergenic non-protein coding RNA 2728
HIGD1C	-0.56081	0.033813	0.033813	-2.2304	Down	HIG1 hypoxia inducible domain family member 1C
MYO7B	-0.56059	0.041943	0.041943	-2.13051	Down	myosin VIIB
OR3A1	-0.55924	0.026476	0.026476	-2.34125	Down	olfactory receptor family 3 subfamily A member 1 (gene/pseudogene)
CD2	-0.55858	0.027018	0.027018	-2.33215	Down	CD2 molecule
CD248	-0.55695	0.037471	0.037471	-2.18305	Down	CD248 molecule
C3orf80	-0.55604	0.020563	0.020563	-2.45323	Down	chromosome 3 open reading frame 80
SIGLEC16	-0.55588	0.029906	0.029906	-2.28635	Down	sialic acid binding Ig like lectin 16 (gene/pseudogene)
ICAM5	-0.55287	0.038001	0.038001	-2.17654	Down	intercellular adhesion molecule 5
LINC02593	-0.55209	0.024742	0.024742	-2.37149	Down	long intergenic non-protein coding RNA 2593
BLNK	-0.54887	0.015616	0.015616	-2.57256	Down	B cell linker
SMOC2	-0.54705	0.035838	0.035838	-2.20365	Down	SPARC related modular calcium binding 2
SLC16A12	-0.54659	0.03348	0.03348	-2.23494	Down	solute carrier family 16 member 12
CD200R1	-0.53901	0.010696	0.010696	-2.73282	Down	CD200 receptor 1
RFX8	-0.53709	0.037009	0.037009	-2.1888	Down	RFX family member 8, lacking RFX DNA binding domain
SLC15A2	-0.5366	0.037165	0.037165	-2.18685	Down	solute carrier family 15 member 2
COLCA1	-0.53394	0.026332	0.026332	-2.34368	Down	colorectal cancer associated 1
FNDC5	-0.53191	0.04585	0.04585	-2.08855	Down	fibronectin type III domain containing 5
TRIM50	-0.53099	0.023565	0.023565	-2.39315	Down	tripartite motif containing 50
HPD	-0.52962	0.030271	0.030271	-2.28086	Down	4-hydroxyphenylpyruvate dioxygenase
DIRAS3	-0.52645	0.042268	0.042268	-2.1269	Down	DIRAS family GTPase 3
TRPV4	-0.52594	0.01629	0.01629	-2.5544	Down	transient receptor potential cation channel subfamily V member 4
PDGFD	-0.52569	0.036484	0.036484	-2.1954	Down	platelet derived growth factor D
LRRC2	-0.52497	0.004795	0.004795	-3.06064	Down	leucine rich repeat containing 2
BAMBI	-0.52363	0.00058	0.00058	-3.87367	Down	BMP and activin membrane bound inhibitor
MRC1	-0.52267	0.008283	0.008283	-2.8389	Down	mannose receptor C-type 1
LIPM	-0.52188	0.032968	0.032968	-2.24199	Down	lipase family member M
FCGR2B	-0.52033	0.028388	0.028388	-2.3099	Down	Fc fragment of IgG receptor IIb
NEXN	-0.51863	2.22E-05	2.22E-05	-5.07043	Down	nexilin F-actin binding protein
CGREF1	-0.51368	0.021369	0.021369	-2.43635	Down	cell growth regulator with EF-hand domain 1
TLR7	-0.51345	0.01367	0.01367	-2.62939	Down	toll like receptor 7
RUNX2	-0.51175	0.015836	0.015836	-2.56656	Down	RUNX family transcription factor 2
NANOS1	-0.51061	0.010494	0.010494	-2.7408	Down	nanos C2HC-type zinc finger 1
CD163L1	-0.51047	0.023747	0.023747	-2.38974	Down	CD163 molecule like 1
RNASE1	-0.50647	0.007879	0.007879	-2.85947	Down	ribonuclease A family member 1, pancreatic
SIGLEC11	-0.50389	0.014684	0.014684	-2.59893	Down	sialic acid binding Ig like lectin 11
KLRD1	-0.5033	0.031927	0.031927	-2.25665	Down	killer cell lectin like receptor D1
COL12A1	-0.50322	0.004436	0.004436	-3.09172	Down	collagen type XII alpha 1 chain
GLIPR2	-0.50158	0.017678	0.017678	-2.51911	Down	GLI pathogenesis related 2
LOX	-0.49993	0.009205	0.009205	-2.7953	Down	lysyl oxidase
LOC339192	-0.4991	0.004991	0.004991	-3.04463	Down	uncharacterized LOC339192
LOC100507477	-0.49658	0.012644	0.012644	-2.66249	Down	uncharacterized LOC100507477
SLC5A1	-0.49615	0.029007	0.029007	-2.30017	Down	solute carrier family 5 member 1
ADARB2	-0.49613	0.047885	0.047885	-2.06793	Down	adenosine deaminase RNA specific B2 (inactive)
HTR1F	-0.49612	0.029137	0.029137	-2.29815	Down	5-hydroxytryptamine receptor 1F
ADRA1B	-0.49337	0.040435	0.040435	-2.14765	Down	adrenoceptor alpha 1B
ADAM12	-0.49149	0.003101	0.003101	-3.23333	Down	ADAM metallopeptidase domain 12
ASAH1	-0.49116	0.010546	0.010546	-2.73872	Down	N-acylsphingosineamidohydrolase 1
F2R	-0.48858	0.00113	0.00113	-3.6225	Down	coagulation factor II thrombin receptor
SCN9A	-0.48853	0.011298	0.011298	-2.70989	Down	sodium voltage-gated channel alpha subunit 9
VNN1	-0.48719	0.045559	0.045559	-2.09156	Down	vanin 1
CLCA2	-0.48349	0.025225	0.025225	-2.36288	Down	chloride channel accessory 2
PALLD	-0.48322	0.021141	0.021141	-2.44107	Down	palladin, cytoskeletal associated protein
FAM234B	-0.48245	0.00126	0.00126	-3.58132	Down	family with sequence similarity 234 member B
TKTL2	-0.47941	0.007788	0.007788	-2.86422	Down	transketolase like 2
IL1RAPL2	-0.47891	0.027206	0.027206	-2.32904	Down	interleukin 1 receptor accessory protein like 2
ALDH1A3	-0.47731	0.03362	0.03362	-2.23302	Down	aldehyde dehydrogenase 1 family member A3
METTL21C	-0.47463	0.049049	0.049049	-2.05649	Down	methyltransferase like 21C
LOXL1	-0.47425	0.001529	0.001529	-3.50725	Down	lysyl oxidase like 1
XKRX	-0.47305	0.047636	0.047636	-2.07042	Down	XK related X-linked
WDR66	-0.47255	0.025331	0.025331	-2.36101	Down	WD repeat domain 66
CRABP2	-0.47169	0.036907	0.036907	-2.19008	Down	cellular retinoic acid binding protein 2
C1QA	-0.46939	0.036047	0.036047	-2.20097	Down	complement C1q A chain
ETV5	-0.46625	0.002936	0.002936	-3.2548	Down	ETS variant transcription factor 5
GPR62	-0.46587	0.03508	0.03508	-2.2135	Down	G protein-coupled receptor 62
LINC00565	-0.46566	0.016548	0.016548	-2.54762	Down	long intergenic non-protein coding RNA 565
CCDC167	-0.46454	0.018942	0.018942	-2.4891	Down	coiled-coil domain containing 167
OTUD7A	-0.46045	0.025299	0.025299	-2.36158	Down	OTU deubiquitinase 7A
RND3	-0.45828	0.001137	0.001137	-3.62035	Down	Rho family GTPase 3
FN1	-0.45811	0.040207	0.040207	-2.15029	Down	fibronectin 1
PCDHA6	-0.45753	0.023652	0.023652	-2.39151	Down	protocadherin alpha 6
DBX2	-0.45586	0.017039	0.017039	-2.53503	Down	developing brain homeobox 2
MAMDC2	-0.45565	0.042331	0.042331	-2.12619	Down	MAM domain containing 2
WDR93	-0.45371	0.02124	0.02124	-2.439	Down	WD repeat domain 93
UCHL1	-0.45268	0.026356	0.026356	-2.34327	Down	ubiquitin C-terminal hydrolase L1
SERPINE2	-0.45078	0.028886	0.028886	-2.30205	Down	serpin family E member 2
AADACL2	-0.44945	0.01659	0.01659	-2.54653	Down	arylacetamidedeacetylase like 2
SERPINB7	-0.4478	0.047833	0.047833	-2.06845	Down	serpin family B member 7
FAM225A	-0.44693	0.011918	0.011918	-2.68743	Down	family with sequence similarity 225 member A
C10orf90	-0.44605	0.015297	0.015297	-2.58142	Down	chromosome 10 open reading frame 90
FBLL1	-0.44578	0.01963	0.01963	-2.47353	Down	fibrillarin like 1
NBPF6	-0.44335	0.021245	0.021245	-2.4389	Down	NBPF member 6
FTMT	-0.44299	0.049479	0.049479	-2.05232	Down	ferritin mitochondrial
LINC01920	-0.44193	0.043385	0.043385	-2.11463	Down	long intergenic non-protein coding RNA 1920
BEND4	-0.44007	0.046768	0.046768	-2.07915	Down	BEN domain containing 4
LINC02338	-0.4399	0.043605	0.043605	-2.11226	Down	long intergenic non-protein coding RNA 2338
HHLA2	-0.43741	0.01909	0.01909	-2.48571	Down	HERV-H LTR-associating 2
HGF	-0.43398	0.013508	0.013508	-2.63446	Down	hepatocyte growth factor
HEXIM1	-0.43356	0.004293	0.004293	-3.10478	Down	HEXIM P-TEFb complex subunit 1
DGKI	-0.43244	0.01747	0.01747	-2.52421	Down	diacylglycerol kinase iota
FILIP1L	-0.43169	0.002395	0.002395	-3.33416	Down	filamin A interacting protein 1 like
EFCAB7	-0.43071	0.023889	0.023889	-2.3871	Down	EF-hand calcium binding domain 7
SLC4A7	-0.43025	0.010957	0.010957	-2.72274	Down	solute carrier family 4 member 7
ZFAT	-0.42877	0.002173	0.002173	-3.37191	Down	zinc finger and AT-hook domain containing
C10orf143	-0.42858	0.008135	0.008135	-2.84633	Down	chromosome 10 open reading frame 143
TNFRSF19	-0.42847	0.037177	0.037177	-2.18671	Down	TNF receptor superfamily member 19
FAM83A	-0.42751	0.039597	0.039597	-2.15742	Down	family with sequence similarity 83 member A
GABRB1	-0.42732	0.035765	0.035765	-2.2046	Down	gamma-aminobutyric acid type A receptor beta1 subunit
NOX4	-0.42605	0.02526	0.02526	-2.36227	Down	NADPH oxidase 4
CCND2	-0.42423	0.021978	0.021978	-2.42397	Down	cyclin D2
THAP10	-0.42349	0.022044	0.022044	-2.42265	Down	THAP domain containing 10
TPTEP1	-0.42268	0.020941	0.020941	-2.44524	Down	TPTE pseudogene 1
HLA-DRB1	-0.42192	0.021662	0.021662	-2.43035	Down	major histocompatibility complex, class II, DR beta 1
LILRB5	-0.42095	0.035206	0.035206	-2.21185	Down	leukocyte immunoglobulin like receptor B5
STAB1	-0.42046	0.028034	0.028034	-2.31555	Down	stabilin 1
NAIP	-0.41965	0.028516	0.028516	-2.30788	Down	NLR family apoptosis inhibitory protein
GALNT16	-0.41849	0.032852	0.032852	-2.2436	Down	polypeptide N-acetylgalactosaminyltransferase 16
WWC1	-0.41823	0.042955	0.042955	-2.11932	Down	WW and C2 domain containing 1
COL3A1	-0.4161	0.012907	0.012907	-2.65376	Down	collagen type III alpha 1 chain
MIR6809	-0.41533	0.042532	0.042532	-2.12397	Down	microRNA 6809
JAZF1	-0.41531	0.007662	0.007662	-2.87091	Down	JAZF zinc finger 1
GALNT7	-0.41474	0.00206	0.00206	-3.39265	Down	polypeptide N-acetylgalactosaminyltransferase 7
SLC30A1	-0.41231	0.044132	0.044132	-2.10659	Down	solute carrier family 30 member 1
SLC24A3	-0.41214	0.004843	0.004843	-3.05668	Down	solute carrier family 24 member 3
IL20RB	-0.41053	0.034294	0.034294	-2.22392	Down	interleukin 20 receptor subunit beta
TMEM200A	-0.41005	0.021657	0.021657	-2.43046	Down	transmembrane protein 200A
LOC100505549	-0.40982	0.023119	0.023119	-2.40162	Down	uncharacterized LOC100505549
HACD4	-0.40883	0.022474	0.022474	-2.41412	Down	3-hydroxyacyl-CoA dehydratase 4
FMOD	-0.40733	0.018574	0.018574	-2.49765	Down	fibromodulin
MARCKS	-0.40702	0.030225	0.030225	-2.28156	Down	myristoylated alanine rich protein kinase C substrate
GLB1	-0.40571	0.002763	0.002763	-3.27859	Down	galactosidase beta 1
CSF1R	-0.40406	0.046599	0.046599	-2.08087	Down	colony stimulating factor 1 receptor
ADGRG6	-0.40323	0.045778	0.045778	-2.0893	Down	adhesion G protein-coupled receptor G6
PIK3R2	-0.40255	0.018858	0.018858	-2.49105	Down	phosphoinositide-3-kinase regulatory subunit 2
PTGFRN	-0.40253	0.007558	0.007558	-2.8765	Down	prostaglandin F2 receptor inhibitor
TACR1	-0.4014	0.022803	0.022803	-2.40771	Down	tachykinin receptor 1
INSM1	-0.40121	0.043164	0.043164	-2.11704	Down	INSM transcriptional repressor 1
CYBB	-0.40065	0.04148	0.04148	-2.13572	Down	cytochrome b-245 beta chain
NRP2	-0.39927	0.007496	0.007496	-2.87988	Down	neuropilin 2
ARRDC4	-0.39869	0.004531	0.004531	-3.08331	Down	arrestin domain containing 4
ASPN	-0.39605	0.018067	0.018067	-2.50967	Down	asporin
CTSW	-0.39553	0.037568	0.037568	-2.18186	Down	cathepsin W
CACNA1C	-0.39553	0.030995	0.030995	-2.27013	Down	calcium voltage-gated channel subunit alpha1 C
SNHG21	-0.39342	0.015072	0.015072	-2.58776	Down	small nucleolar RNA host gene 21
ITGAV	-0.39277	0.001011	0.001011	-3.6648	Down	integrin subunit alpha V
LUM	-0.39237	0.048966	0.048966	-2.0573	Down	lumican
TPM2	-0.39197	0.024526	0.024526	-2.3754	Down	tropomyosin 2
DAB2	-0.39195	4.44E-05	4.44E-05	-4.81792	Down	DAB adaptor protein 2
GGTA1P	-0.38792	0.018167	0.018167	-2.50726	Down	glycoprotein alpha-galactosyltransferase 1, pseudogene
MIMT1	-0.38785	0.036712	0.036712	-2.19253	Down	MER1 repeat containing imprinted transcript 1
OR9Q2	-0.38734	0.03162	0.03162	-2.26104	Down	olfactory receptor family 9 subfamily Q member 2
FAM20A	-0.38702	0.036624	0.036624	-2.19364	Down	FAM20A golgi associated secretory pathway pseudokinase
PMEPA1	-0.38651	0.009631	0.009631	-2.77655	Down	prostate transmembrane protein, androgen induced 1
SAMSN1	-0.38607	0.027046	0.027046	-2.33168	Down	SAM domain, SH3 domain and nuclear localization signals 1
TAFA2	-0.38579	0.046957	0.046957	-2.07723	Down	TAFA chemokine like family member 2
SLC6A7	-0.38559	0.04736	0.04736	-2.07317	Down	solute carrier family 6 member 7
CKMT2-AS1	-0.38548	0.045796	0.045796	-2.08911	Down	CKMT2 antisense RNA 1
MPEG1	-0.38475	0.032517	0.032517	-2.24829	Down	macrophage expressed 1
C19orf18	-0.38407	0.04723	0.04723	-2.07448	Down	chromosome 19 open reading frame 18
KLHL29	-0.38309	0.035953	0.035953	-2.20218	Down	kelch like family member 29
CERCAM	-0.38247	0.019646	0.019646	-2.47319	Down	cerebral endothelial cell adhesion molecule
ETV1	-0.38153	0.029051	0.029051	-2.29948	Down	ETS variant transcription factor 1
BDKRB2	-0.38151	0.040133	0.040133	-2.15114	Down	bradykinin receptor B2
GRHL1	-0.37827	0.012852	0.012852	-2.65557	Down	grainyhead like transcription factor 1
OTULINL	-0.37674	0.028403	0.028403	-2.30967	Down	OTU deubiquitinase with linear linkage specificity like
RGL1	-0.37613	0.000119	0.000119	-4.45727	Down	ral guanine nucleotide dissociation stimulator like 1
RPL13P5	-0.37585	0.048146	0.048146	-2.06535	Down	ribosomal protein L13 pseudogene 5
ZNF488	-0.37547	0.047689	0.047689	-2.06988	Down	zinc finger protein 488
SLC25A14	-0.37434	0.008636	0.008636	-2.82168	Down	solute carrier family 25 member 14
LINC01140	-0.37257	0.004509	0.004509	-3.08519	Down	long intergenic non-protein coding RNA 1140
UGDH	-0.37133	0.02146	0.02146	-2.43449	Down	UDP-glucose 6-dehydrogenase
ENC1	-0.37101	0.018286	0.018286	-2.50442	Down	ectodermal-neural cortex 1
RTL9	-0.36928	0.047629	0.047629	-2.07048	Down	retrotransposon Gag like 9
IQGAP2	-0.36865	0.037295	0.037295	-2.18524	Down	IQ motif containing GTPase activating protein 2
ALDH1L2	-0.36654	0.000623	0.000623	-3.84689	Down	aldehyde dehydrogenase 1 family member L2
GPR34	-0.36515	0.039956	0.039956	-2.15321	Down	G protein-coupled receptor 34
GRAMD1B	-0.36456	0.044696	0.044696	-2.1006	Down	GRAM domain containing 1B
MAN1A1	-0.36373	0.006066	0.006066	-2.96604	Down	mannosidase alpha class 1A member 1
RABIF	-0.36301	0.002326	0.002326	-3.34563	Down	RAB interacting factor
TEC	-0.36224	0.005923	0.005923	-2.97566	Down	tec protein tyrosine kinase
FICD	-0.36185	0.000247	0.000247	-4.19042	Down	FIC domain containing
PLXDC1	-0.36111	0.037452	0.037452	-2.18329	Down	plexin domain containing 1
C1orf146	-0.36015	0.038798	0.038798	-2.1669	Down	chromosome 1 open reading frame 146
DYNC1I1	-0.35903	0.043271	0.043271	-2.11587	Down	dynein cytoplasmic 1 intermediate chain 1
MFAP4	-0.35865	0.014841	0.014841	-2.59438	Down	microfibril associated protein 4
FRMD4B	-0.35817	0.040289	0.040289	-2.14934	Down	FERM domain containing 4B
P2RY13	-0.35797	0.029676	0.029676	-2.28985	Down	purinergic receptor P2Y13
HRH1	-0.35784	0.044282	0.044282	-2.10499	Down	histamine receptor H1
FBN1	-0.35729	0.008031	0.008031	-2.85159	Down	fibrillin 1
COL5A2	-0.35639	0.000402	0.000402	-4.01019	Down	collagen type V alpha 2 chain
ALX4	-0.35556	0.024298	0.024298	-2.37955	Down	ALX homeobox 4
CCDC80	-0.35542	0.001101	0.001101	-3.63236	Down	coiled-coil domain containing 80
SNCAIP	-0.35503	0.013865	0.013865	-2.62339	Down	synuclein alpha interacting protein
IKBIP	-0.354	0.02324	0.02324	-2.3993	Down	IKBKB interacting protein
BICC1	-0.35252	0.004878	0.004878	-3.05381	Down	BicC family RNA binding protein 1
CAMK2N1	-0.34899	0.047228	0.047228	-2.0745	Down	calcium/calmodulin dependent protein kinase II inhibitor 1
TMEM67	-0.34689	0.024989	0.024989	-2.36708	Down	transmembrane protein 67
LIMCH1	-0.34449	0.005071	0.005071	-3.0382	Down	LIM and calponin homology domains 1
GNG2	-0.34436	0.017366	0.017366	-2.52679	Down	G protein subunit gamma 2
TRAM2	-0.34185	0.001914	0.001914	-3.42104	Down	translocation associated membrane protein 2
VLDLR	-0.34162	0.011677	0.011677	-2.69604	Down	very low density lipoprotein receptor
TTYH2	-0.33982	0.049674	0.049674	-2.05044	Down	tweety family member 2
P4HA2	-0.33879	0.00227	0.00227	-3.35509	Down	prolyl 4-hydroxylase subunit alpha 2
FER1L6	-0.33814	0.027267	0.027267	-2.32803	Down	fer-1 like family member 6
GEM	-0.33728	0.005769	0.005769	-2.98636	Down	GTP binding protein overexpressed in skeletal muscle
RCN3	-0.33692	0.02923	0.02923	-2.2967	Down	reticulocalbin 3
IMPAD1	-0.33553	0.005228	0.005228	-3.02597	Down	inositol monophosphatase domain containing 1
SESN1	-0.33492	0.006934	0.006934	-2.91171	Down	sestrin 1
GPR137B	-0.33349	0.036019	0.036019	-2.20133	Down	G protein-coupled receptor 137B
SPARC	-0.33102	0.007711	0.007711	-2.86828	Down	secreted protein acidic and cysteine rich
ZDHHC20	-0.33048	0.021857	0.021857	-2.4264	Down	zinc finger DHHC-type containing 20
CPXM2	-0.32867	0.027253	0.027253	-2.32826	Down	carboxypeptidase X, M14 family member 2
RRN3P2	-0.32859	0.018624	0.018624	-2.49648	Down	RRN3 homolog, RNA polymerase I transcription factor pseudogene 2
SEMA3C	-0.32851	0.017989	0.017989	-2.51154	Down	semaphorin 3C
DACT1	-0.32813	0.03652	0.03652	-2.19495	Down	dishevelled binding antagonist of beta catenin 1
SPDYE17	-0.32802	0.04574	0.04574	-2.08968	Down	speedy/RINGO cell cycle regulator family member E17
LINC01963	-0.32754	0.012373	0.012373	-2.67163	Down	long intergenic non-protein coding RNA 1963
GIN1	-0.32533	0.038359	0.038359	-2.17219	Down	gypsy retrotransposonintegrase 1
HECTD2	-0.32446	0.036485	0.036485	-2.19539	Down	HECT domain E3 ubiquitin protein ligase 2
PTGER4	-0.32374	0.014657	0.014657	-2.5997	Down	prostaglandin E receptor 4
PRNP	-0.32047	0.00413	0.00413	-3.12014	Down	prion protein
MAP 1B	-0.32035	0.015796	0.015796	-2.56765	Down	microtubule associated protein 1B
GAS1RR	-0.31983	0.042327	0.042327	-2.12624	Down	GAS1 adjacent regulatory RNA
ZNF107	-0.31823	0.00885	0.00885	-2.81159	Down	zinc finger protein 107
FSTL3	-0.31787	0.020675	0.020675	-2.45085	Down	follistatin like 3
PKD1L3	-0.31687	0.0431	0.0431	-2.11773	Down	polycystin 1 like 3, transient receptor potential channel interacting
PAG1	-0.31685	0.019249	0.019249	-2.48211	Down	phosphoprotein membrane anchor with glycosphingolipidmicrodomains 1
TMPPE	-0.31551	0.000808	0.000808	-3.74957	Down	transmembrane protein with metallophosphoesterase domain
FRRS1L	-0.31367	0.047263	0.047263	-2.07416	Down	ferric chelate reductase 1 like
WDR78	-0.31163	0.036163	0.036163	-2.19948	Down	WD repeat domain 78
OLFML2B	-0.31035	0.034022	0.034022	-2.22757	Down	olfactomedin like 2B
SH3RF3	-0.31019	0.019582	0.019582	-2.4746	Down	SH3 domain containing ring finger 3
SLC38A6	-0.30913	0.045079	0.045079	-2.09657	Down	solute carrier family 38 member 6
GBP4	-0.30841	0.017695	0.017695	-2.51868	Down	guanylate binding protein 4
ZNF678	-0.30546	0.016314	0.016314	-2.55377	Down	zinc finger protein 678
CYTOR	-0.30436	0.014748	0.014748	-2.59705	Down	cytoskeleton regulator RNA
TDRD15	-0.30397	0.022379	0.022379	-2.416	Down	tudor domain containing 15
ARHGAP18	-0.30123	0.003517	0.003517	-3.18376	Down	Rho GTPase activating protein 18
INSYN2A	-0.29921	0.007796	0.007796	-2.86381	Down	inhibitory synaptic factor 2A
SLC2A10	-0.29836	0.011493	0.011493	-2.7027	Down	solute carrier family 2 member 10
TENM1	-0.29763	0.023018	0.023018	-2.40355	Down	teneurintransmembrane protein 1
COLEC12	-0.29666	0.027788	0.027788	-2.31953	Down	collectin subfamily member 12
MTSS1	-0.29656	0.021939	0.021939	-2.42476	Down	MTSS I-BAR domain containing 1
FSTL1	-0.29568	0.004621	0.004621	-3.07542	Down	follistatin like 1
KLF10	-0.29395	0.015541	0.015541	-2.57463	Down	Kruppel like factor 10
UGP2	-0.29329	0.044865	0.044865	-2.09882	Down	UDP-glucose pyrophosphorylase 2
SLC9A9	-0.29287	0.005573	0.005573	-3.00025	Down	solute carrier family 9 member A9
LPAR1	-0.29287	0.008363	0.008363	-2.83493	Down	lysophosphatidic acid receptor 1
GABRG1	-0.29068	0.037398	0.037398	-2.18397	Down	gamma-aminobutyric acid type A receptor gamma1 subunit
PDLIM1	-0.29033	0.018561	0.018561	-2.49795	Down	PDZ and LIM domain 1
EEA1	-0.29004	0.01712	0.01712	-2.53298	Down	early endosome antigen 1
RUNX1	-0.28986	0.039414	0.039414	-2.15957	Down	RUNX family transcription factor 1
MTHFD1L	-0.28913	0.046147	0.046147	-2.08549	Down	methylenetetrahydrofolate dehydrogenase (NADP+ dependent) 1 like
MIR4435-2HG	-0.28849	0.007052	0.007052	-2.90483	Down	MIR4435-2 host gene
FAM118B	-0.28811	0.005667	0.005667	-2.99355	Down	family with sequence similarity 118 member B
CASP6	-0.28807	0.038673	0.038673	-2.16841	Down	caspase 6
MAN2A1	-0.28713	0.008895	0.008895	-2.80947	Down	mannosidase alpha class 2A member 1
LINC02693	-0.28672	0.036645	0.036645	-2.19337	Down	long intergenic non-protein coding RNA 2693
C2CD6	-0.28663	0.049977	0.049977	-2.04753	Down	C2 calcium dependent domain containing 6
SOCS5	-0.28573	0.039472	0.039472	-2.15889	Down	suppressor of cytokine signaling 5
MTF1	-0.28509	0.039683	0.039683	-2.15641	Down	metal regulatory transcription factor 1
ATRN	-0.28445	2.25E-05	2.25E-05	-5.06531	Down	attractin
ENTHD1	-0.28253	0.03304	0.03304	-2.24099	Down	ENTH domain containing 1
ZNF569	-0.28241	0.032207	0.032207	-2.25265	Down	zinc finger protein 569
SYNC	-0.28167	0.026701	0.026701	-2.33744	Down	syncoilin, intermediate filament protein

### Gene ontology and pathway enrichment analyses

DEGs were divided into up regulated genes and down regulated genes. GO and REACTOME pathway enrichment analysis were conducted for DEGs. Results of GO categories were presented by functional groups, which include BP, CC, and MF, and are listed in Table 3. In group BP, up regulated genes enriched in regulation of ion transmembrane transport and oxoacid metabolic process, while the down regulated genes enriched in cell adhesion and response to endogenous stimulus. For group CC, up regulated genes enriched in intrinsic component of plasma membrane and mitochondrion, while down regulated genes enriched in integral component of plasma membrane and supra molecular fiber. In addition, GO results of group MF showed that up regulated genes enriched in transferase activity, transferring phosphorus-containing groups and transporter activity and down regulated genes enriched in signaling receptor binding and molecular transducer activity. Several significant enriched pathways were acquired through REACTOME pathway analysis (Table 4). The enriched pathways for up regulated genes included integration of energy metabolism and neuronal system, while, down regulated genes enriched in extracellular matrix organization and GPCR ligand binding.

**Table 3:** The enriched GO terms of the up and down regulated differentially expressed genes

GO ID	CATEGORY	GO Name	P Value	FDR B&H	FDR B&Y	Bonferroni	Gene Count	Gene
**Up regulated genes**								
GO:0034765	BP	regulation of ion transmembrane transport	2.49E-04	9.88E-02	8.85E-01	1.00E+00	21	KCNE5,RRAD,SLC9A3R2,SLC9A3R1,NRXN1,RAPGEF3,SCN4A,SHANK3,CRHR1,SHANK2,SPHK2,PSEN2,PTPN3,VAMP2,CACNA1A,KCNE2,CACNB2,TMC2,CASQ2,KCNK7,EDNRA
GO:0043436	BP	oxoacid metabolic process	9.54E-04	1.64E-01	1.00E+00	1.00E+00	35	ACADS,ACAT2,GGT6,PFKFB3,PGAM2,ADH1B,CKB,FASN,PLIN5,MPST,SLC6A8,CSPG4,EGFLAM,PRODH,CYP2E1,SREBF1,GCK,ALDH1L1,TYSND1,ACSS2,ITIH1,LDHD,ADHFE1,GK,SULT1C2,SPHK1,GLUL,GPT2,CACNA1A,ANKRD23,MLXIPL,GCAT,AASS,HADH,DCXR
GO:0031226	CC	intrinsic component of plasma membrane	3.56E-04	2.11E-02	1.44E-01	1.90E-01	48	EPHB4,HLA-DQA1,KCNE5,CD320,REEP2,NRXN1,SCN4A,NPC1L1,RAB26,ALK,CNTFR,ADCY4,ACVR1C,SHANK3,CRHR1,SLC2A4,SLC6A8,SHANK2,GPIHBP1,PLPP2,SLC26A7,CSPG4,EBP,FUT1,SLC4A4,SLC16A13,PSEN2,ADGRB3,FGFRL1,SLC30A3,SEMA4C,VAMP2,CA4,CACNA1A,KCNE2,DDR1,CACNB2,STBD1,TMC2,PCDHGC4,SLC19A3,SLC35G2,DLL1,EDA,KCNK7,SYT7,EDNRA,TGM2
GO:0005739	CC	mitochondrion	2.66E-03	6.74E-02	4.62E-01	1.00E+00	46	HDHD3,ACADS,ACAT2,ESR2,GLYCTK,RDH13,YJEFN3,CKB,PCBD2,FASN,PLIN5,MPST,ADCK5,LGALS12,SLC25A10,MRPL11,PRODH,SPHK2,CYP2E1,MYOC,GCK,ALDH1L1,STOX1,ACSS2,POLR1G,LDHD,ADHFE1,GK,BOK,COQ8A,GLUL,PXMP2,MAIP1,GPT2,MGARP,TDRKH,DEPP1,ECHDC3,SLC25A22,TGM2,GCAT,AASS,SLC25A52,HADH,TMEM143,TP73
GO:0016772	MF	transferase activity, transferring phosphorus-containing groups	1.80E-04	4.04E-02	2.98E-01	1.62E-01	52	CDK20,EPHB4,GPHN,CDKN2C,DAPK2,GLYCTK,CARD10,PFKFB3,PGAM2,MAP 3K5,CKB,CISH,SLC9A3R1,NRXN1,PI4KAP1,ALPK3,TAB1,ALK,MOCS1,PKN3,ANKK1,ADCY4,ADCK5,WNT11,ACVR1C,AATK,NEK8,CSPG4,PNCK,SPHK2,PSEN2,IRAK2,FGFRL1,GCK,STOX1,POLR1G,RASIP1,GK,COQ8A,SPHK1,CCNA1,GNG3,CCNJL,DDR1,POLM,MKNK2,WASHC1,SRCIN1,RGS3,EDNRA,MLXIPL,TP73
GO:0005215	MF	transporter activity	2.72E-03	1.37E-01	1.00E+00	1.00E+00	40	KCNE5,RRAD,CD320,SLC9A3R1,NRXN1,RAPGEF3,SCN4A,NPC1L1,SVOP,APOB,SHROOM2,SHANK3,CRHR1,SLC2A4,SLC6A8,SHANK2,GPIHBP1,SLC26A7,EBP,SLC25A10,SLC4A4,SLC2A11,SPHK2,SLC16A13,AZGP1,CPLX1,SLC30A3,APOM,PTPN3,VAMP2,CACNA1A,KCNE2,CACNB2,TMC2,INAFM1,SLC19A3,TMEM63C,CASQ2,KCNK7,SLC25A22
**Down regulated genes**								
GO:0046649	BP	lymphocyte activation	1.37E-15	5.40E-12	4.78E-11	5.40E-12	40	HLA-DMB,HLA-DPA1,SPTA1,BCL11A,ITGA4,FGL2,CXCR5,FCRL1,PTPRC,ZC3H12D,FBXO7,CCR7,NOTCH2,TCF7,IKZF3,POLM,CAMK4,POU2F2,BTLA,CR1,CBLB,SLC4A1,TFRC,BCL11B,CD4,CD5,CD6,MS4A1,CD22,CD27,CD44,TNFRSF13C,TNFRSF13B,CD79A,RORA,ATM,LY9,LEF1,IL7R,NCKAP1L
GO:0010941	BP	regulation of cell death	9.53E-05	6.03E-03	5.34E-02	3.75E-01	40	STK17B,OBSCN,ITGA4,NR4A1,STRADB,BNIP3L,PTPRC,BTG1,TMC8,FBXO7,PLAGL2,CCR7,NOTCH2,TCF7,IKZF3,OGT,CAMK1D,BMF,NLRP1,GRINA,OPA1,EEF1A1,TXNIP,BCL11B,IGF2R,CSF1R,CD27,NEURL1,CD44,LRP1,ATM,HBA1,HBA2,HBB,CTSB,LEF1,RPL10,IL7R,SORL1,NCKAP1L
GO:0009986	CC	cell surface	2.02E-09	3.10E-07	2.08E-06	9.30E-07	35	ADAM19,HLA-DPA1,HLA-DRA,ITGA4,CXCR5,FCRL1,PTPRC,CIITA,MRC2,CCR7,NOTCH2,SEMA7A,BTLA,CR1,SLC4A1,TFRC,CD4,CD5,IGF2R,CD6,CSF1R,MS4A1,CLEC17A,CD22,CD27,GYPA,VCAN,CD44,CLEC2D,TNFRSF13C,TNFRSF13B,CD79A,LY9,CTSB,IL7R
GO:0031226	CC	intrinsic component of plasma membrane	5.35E-07	4.11E-05	2.76E-04	2.47E-04	43	HLA-DPA1,HLA-DRA,SPTA1,SLC38A5,ITGA4,SLC38A1,CXCR5,PTPRC,SLC24A4,TMC8,NOTCH2,VIPR1,TRABD2A,SEMA7A,BTLA,SLC7A6,CR1,MCOLN1,SLC2A1,TSPAN5,RHAG,SLC4A1,TFRC,CELSR1,CD4,CD5,IGF2R,CD6,CSF1R,P2RX5,MS4A1,SLC14A1,CD22,CD27,GYPA,GYPB,CD44,CLEC2D,LRP1,TNFRSF13B,ATP2B1,SORL1,NCKAP1L
GO:0016772	MF	transferase activity, transferring phosphorus-containing groups	9.72E-04	4.32E-02	3.09E-01	6.92E-01	35	ZBTB20,RPS6KA5,STK17B,OBSCN,TENT5C,TTN,PIK3IP1,BLK,STRADB,PTPRC,CIITA,FBXO7,CCR7,DYRK2,OGT,CAMK1D,CTC1,POLM,CAMK4,DUSP2,AAK1,CBLB,SLC4A1,EEF1A1,RMRP,CD4,IGF2R,CSF1R,NEURL1,CD44,LRP1,ATM,SERINC5,SORL1,NCKAP1L
GO:0008144	MF	drug binding	1.15E-02	1.55E-01	1.00E+00	1.00E+00	30	ABCA2,RPS6KA5,STK17B,OBSCN,TTN,BLK,STRADB,CIITA,ALAS2,ABCD2,DYRK2,DNHD1,ACSL6,CAMK1D,EP400,ATP13A1,CAMK4,AAK1,NLRP1,EEF1A1,DNAH1,CSF1R,P2RX5,ATM,HBA1,HBA2,HBM,HBB,ATP2B1,EPB42

Biological Process (BP), Cellular Component (CC) and Molecular Functions (MF)

**Table 4:** The enriched pathway terms of the up and down regulated differentially expressed genes

Pathway ID	Pathway Name	P-value	FDR B&H	FDR B&Y	Bonferroni	Gene Count	Gene
**Up regulated genes**							
1269340	Hemostasis	4.28E-06	7.47E-04	5.11E-03	2.24E-03	22	GNG8,TRPC6,CEACAM6,CABLES1,SERPINE2,FGA,KCNMB1,FGB,HRG,PLA2G4A,MMP1,FGG,VEGFC,GRB14,APOB,APOH,ZFPM2,TGFB3,ORM1,TIMP1,CD63,PRKAR1B
1269741	Cell Cycle	8.91E-04	2.12E-02	1.45E-01	4.67E-01	17	CETN2,MND1,MCM10,GINS1,TYMS,H2BC17,H4C14,BUB1,H2BC11,UBE2C,H4C15,CENPU,E2F1,CCNB1,DMC1,CENPI,H2AJ
1269507	Signaling by Rho GTPases	1.14E-02	8.42E-02	5.76E-01	1.00E+00	11	PPP1R14A,TAX1BP3,H2BC17,H4C14,BUB1,WASF1,H2BC11,H4C15,CENPU,CENPI,H2AJ
1269203	Innate Immune System	7.89E-02	2.91E-01	1.00E+00	1.00E+00	21	CEACAM6,DEFA1,GLA,HP,VNN1,FGA,FGB,FGG,WASF1,PGLYRP1,APOB,RET,LCN2,POLR3G,DERA,ORM1,MS4A3,CYSTM1,CD63,PRKAR1B,MCEMP1
1270001	Metabolism of lipids and lipoproteins	1.52E-01	3.82E-01	1.00E+00	1.00E+00	13	ACER2,ACOT7,ME1,GLA,SPHK1,PLA2G4A,FHL2,PLAAT1,G0S2,THEM5,APOA2,APOB,SMPD1
1268677	Metabolism of proteins	3.37E-01	5.01E-01	1.00E+00	1.00E+00	21	GNG8,CETN2,FN3K,H2AC13,H2BC17,VNN1,SPHK1,H4C14,MITF,FGA,MMP1,RAB32,METTL22,DYNC1I1,H2BC11,ADAMTS1,UBE2C,H4C15,ADAMTS5,IGFBP2,AOPEP
**Down regulated genes**							
1269171	Adaptive Immune System	1.59E-03	8.64E-02	5.87E-01	7.96E-01	20	HLA-DMB,HLA-DPA1,HLA-DRA,TNRC6B,ITGA4,NR4A1,BLK,KLHL3,PTPRC,MRC2,FBXO7,RNF213,BTLA,CBLB,CD4,CD22,CLEC2D,CD79A,CTSB,RNF182
1269903	Transmembrane transport of small molecules	1.39E-02	1.95E-01	1.00E+00	1.00E+00	15	ABCA2,SLC38A5,SLC38A1,SLC24A4,ABCD2,GNG7,ATP13A1,SLC7A6,MCOLN1,SLC2A1,RHAG,SLC4A1,TFRC,SLC14A1,ATP2B1
1269203	Innate Immune System	8.82E-02	4.76E-01	1.00E+00	1.00E+00	21	RPS6KA5,SPTA1,TNRC6B,NR4A1,ADA2,FGL2,PTPRC,CAMK4,DUSP2,NLRP1,CNKSR2,CR1,EEF1A1,TXNIP,EEF2,CD4,IGF2R,CD44,HBB,CTSB,NCKAP1L
1269876	Vesicle-mediated transport	1.54E-01	5.50E-01	1.00E+00	1.00E+00	11	SPTA1,SEC16A,COL7A1,AAK1,TFRC,CD4,IGF2R,LRP1,HBA1,HBA2,HBB
1268854	Disease	3.21E-01	6.43E-01	1.00E+00	1.00E+00	12	RPL23A,RPL27A,RPL37,NR4A1,NOTCH2,CNKSR2,EEF2,CD4,VCAN,NEURL1,RPL13A,RPL10
1268677	Metabolism of proteins	5.53E-01	7.60E-01	1.00E+00	1.00E+00	19	RPL23A,RPL27A,RPL37,SPTA1,MGAT4A,NEU3,ADA2,TUBB1,YOD1,SEC16A,GNG7,KLK1,COL7A1,OGT,EEF1A1,EEF2,RPL13A,RPL10,SORL1

### PPI network construction and module analysis

**Fig. 3:**
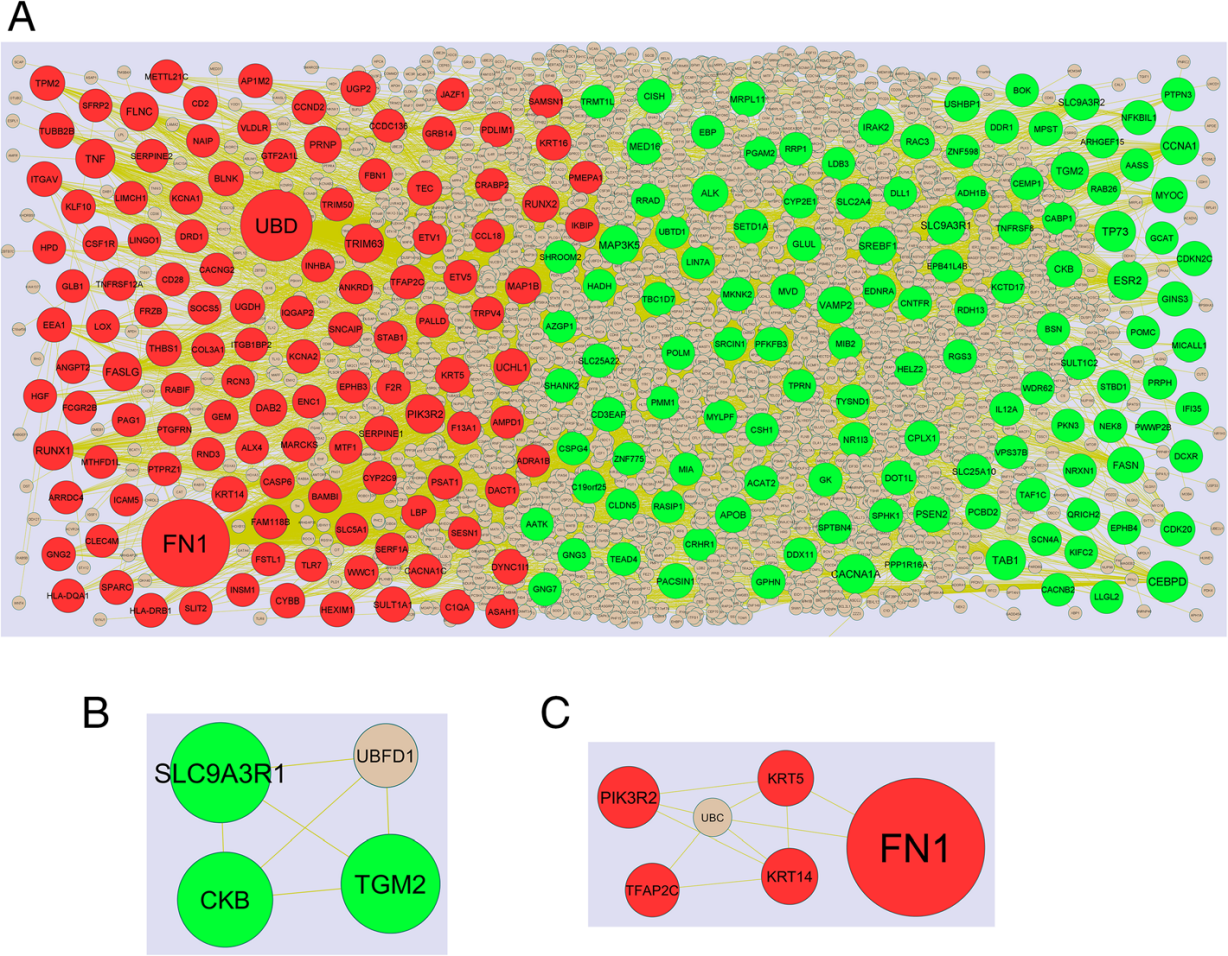
PPI network and the most significant modules of DEGs. **a** The PPI network of DEGs was constructed using Cytoscape **b** The most significant module was obtained from PPI network with 4 nodes and 6 edges for up regulated genes **c** The most significant module was obtained from PPI network with 6 nodes and 10 edges for down regulated genes. Up regulated genes are marked in green; down regulated genes are marked in red

PPI network complex consisted of 3648 nodes and 6305 edges, wherein node and edge represented gene and interaction between genes (Fig.3a). Moreover, CEBPD, TP73, ESR2, TAB1, MAP 3K5, FN1, UBD, RUNX1, PIK3R2 and TNF were identified as hub genes and are listed in Table 5. In addition, module analysis was conducted to detect the highly connected regions of PPI network, and two significant modules were identified (Fig.3b and Fig.3c). Further GO and pathway enrichment analysis revealed that genes in these modules were mostly implicated in regulation of ion transmembrane transport, oxoacid metabolic process, intrinsic component of plasma membrane, extracellular matrix organization and supra molecular fiber.

**Table 5:** Topology table for up and down regulated genes

Regulation	Node	Degree	Betweenness	Stress	Closeness
Up	CEBPD	114	0.046829	7961100	0.330434
Up	TP73	111	0.034635	8777680	0.328559
Up	ESR2	101	0.034485	6488550	0.337342
Up	TAB1	99	0.041885	3759452	0.362813
Up	MAP 3K5	92	0.030461	6831818	0.331998
Up	CACNA1A	91	0.035636	5405420	0.325712
Up	CCNA1	80	0.02152	6576136	0.326587
Up	SLC9A3R1	77	0.024066	3777376	0.332877
Up	SREBF1	70	0.01966	4888424	0.327673
Up	TGM2	69	0.023349	3331196	0.360768
Up	VAMP2	59	0.021393	4433628	0.327614
Up	FASN	57	0.012258	2145144	0.352708
Up	PSEN2	53	0.018322	2705190	0.32389
Up	CKB	51	0.012369	2056678	0.335511
Up	ALK	51	0.013768	1544390	0.292392
Up	MED16	46	0.014057	2049900	0.326909
Up	SLC9A3R2	45	0.013157	2184316	0.317821
Up	APOB	43	0.012557	2206980	0.322002
Up	MYOC	42	0.012184	1022522	0.322658
Up	IRAK2	41	0.012813	1784830	0.321293
Up	SLC2A4	40	0.011976	1766080	0.322601
Up	USHBP1	36	0.015448	3575428	0.248925
Up	MRPL11	34	0.010599	1753194	0.322287
Up	CPLX1	33	0.009034	742112	0.287437
Up	PACSIN1	33	0.010298	664252	0.278015
Up	PCBD2	32	0.010339	2852002	0.275204
Up	SLC25A10	30	0.009439	1406902	0.31647
Up	DOT1L	29	0.007619	3660752	0.26943
Up	CDK20	28	0.007513	1387276	0.314207
Up	ACAT2	28	0.007324	722288	0.326091
Up	CDKN2C	28	0.006528	1250162	0.316004
Up	CISH	27	0.005203	494888	0.295879
Up	GINS3	27	0.00745	1311612	0.3176
Up	MVD	26	0.008346	1085242	0.313613
Up	NR1I3	24	0.004449	1369088	0.285591
Up	RAC3	23	0.005847	909650	0.317324
Up	SETD1A	22	0.006802	741452	0.319296
Up	GLUL	21	0.006551	455932	0.338689
Up	NFKBIL1	20	0.007285	2695372	0.255984
Up	CD3EAP	19	0.004472	455916	0.289697
Up	SHANK2	19	0.00448	1859670	0.264621
Up	GPHN	18	0.005963	580752	0.315157
Up	PPP1R16A	17	0.006189	3748574	0.241315
Up	CYP2E1	17	0.00535	689500	0.318654
Up	IL12A	17	0.003913	940482	0.270248
Up	TAF1C	16	0.004869	576732	0.316965
Up	MPST	15	0.004427	490670	0.315703
Up	CNTFR	15	0.004392	3618160	0.24549
Up	DDR1	15	0.00321	479586	0.317379
Up	MKNK2	14	0.002033	289430	0.322116
Up	KCTD17	13	0.003492	916690	0.244208
Up	RGS3	13	0.001735	620256	0.320982
Up	SPHK1	13	0.002877	655002	0.317655
Up	PRPH	13	0.003388	599412	0.319436
Up	ADH1B	13	0.001294	370206	0.275682
Up	SLC25A22	13	0.003141	530858	0.314776
Up	MIB2	12	0.002205	375196	0.315185
Up	ZNF598	12	0.004687	393930	0.312779
Up	GK	12	0.003466	415618	0.314994
Up	RRAD	12	0.0025	148574	0.282166
Up	PFKFB3	12	0.001355	246878	0.314072
Up	SPTBN4	12	0.004741	379826	0.313909
Up	PTPN3	12	0.001814	952130	0.257957
Up	LIN7A	11	0.004011	512222	0.31796
Up	VPS37B	11	0.00232	329104	0.31372
Up	TRMT1L	11	0.002348	529928	0.319184
Up	EDNRA	11	0.001735	273128	0.314885
Up	TBC1D7	10	0.002122	358246	0.3176
Up	AZGP1	10	0.002218	255470	0.31337
Up	WDR62	10	0.001673	347176	0.314912
Up	NRXN1	10	0.003845	1972864	0.226227
Up	CLDN5	9	0.002871	301080	0.313343
Up	TYSND1	9	0.003301	1096284	0.204234
Up	NEK8	9	0.001286	501688	0.317158
Up	MICALL1	9	0.006015	346680	0.317103
Up	TNFRSF8	8	0.001683	558578	0.221218
Up	DDX11	8	0.002534	239432	0.313964
Up	CRHR1	8	0.002228	518378	0.23728
Up	QRICH2	8	0.002787	388592	0.238787
Up	TEAD4	8	0.002413	296338	0.312993
Up	CSPG4	8	0.001645	199988	0.313289
Up	CABP1	8	0.001378	82156	0.281578
Up	SULT1C2	8	0.001233	207860	0.315676
Up	GNG7	8	0.001801	266812	0.312779
Up	HADH	7	0.001386	314576	0.318043
Up	EBP	7	0.002552	384196	0.317186
Up	CSH1	7	8.48E-05	30798	0.243572
Up	C19orf25	7	0.001712	1024492	0.232753
Up	AASS	7	5.38E-04	163226	0.314804
Up	POLM	6	0.001104	522630	0.201203
Up	BSN	6	0.001531	145360	0.312457
Up	KIFC2	6	0.001203	58718	0.27345
Up	POMC	6	0.002233	811808	0.234609
Up	DCXR	6	0.001279	220510	0.31658
Up	EPHB4	6	8.00E-04	275102	0.317572
Up	RAB26	6	0.002046	260892	0.31658
Up	UBTD1	6	0.001963	183788	0.314586
Up	GNG3	6	8.77E-05	19382	0.228452
Up	SRCIN1	6	1.34E-04	36856	0.257593
Up	DLL1	6	0.001102	106292	0.313074
Up	IFI35	6	0.001499	178126	0.313666
Up	EPB41L4B	6	5.42E-04	276788	0.316497
Up	RDH13	5	0.001833	134020	0.312404
Up	SHROOM2	5	3.34E-05	23708	0.250636
Up	PGAM2	5	3.21E-04	99124	0.313101
Up	RASIP1	5	0.001351	121434	0.312404
Up	LDB3	5	5.77E-05	11670	0.240694
Up	STBD1	5	5.68E-04	91162	0.211163
Up	MYLPF	5	0.001107	295946	0.221057
Up	ZNF775	5	0.00111	426814	0.230721
Up	BOK	5	0.001655	134510	0.312404
Up	ARHGEF15	5	0.001681	719112	0.215939
Up	RRP1	5	6.51E-04	120052	0.314749
Up	LLGL2	5	0.001716	131528	0.312404
Up	PKN3	5	0.002193	149884	0.312404
Up	AATK	5	0.001114	414048	0.231497
Up	HELZ2	5	4.97E-04	113146	0.312752
Up	TPRN	4	5.53E-04	316590	0.229602
Up	PMM1	4	6.74E-04	50432	0.317324
Up	SCN4A	4	0.001101	106362	0.254501
Up	MIA	2	0	0	0.289951
Up	GCAT	1	0	0	0.265816
Up	CACNB2	1	0	0	0.241779
Up	PWWP2B	1	0	0	0.265816
Up	CEMP1	1	0	0	0.265816
Down	FN1	689	0.324012	1.05E+08	0.403385
Down	UBD	502	0.200346	87511340	0.362021
Down	RUNX1	96	0.029748	6112420	0.328351
Down	PIK3R2	91	0.025749	6760118	0.337529
Down	TNF	91	0.029868	5372816	0.339382
Down	TRIM63	88	0.033338	4601374	0.331545
Down	FASLG	83	0.03072	3652094	0.356361
Down	FLNC	77	0.027425	3582900	0.356257
Down	DAB2	63	0.01646	2823298	0.354974
Down	PRNP	61	0.022352	8130382	0.290089
Down	RUNX2	57	0.013307	3614466	0.32323
Down	UCHL1	54	0.013451	2855314	0.328973
Down	MAP 1B	45	0.011916	2421086	0.323574
Down	KRT14	44	0.006308	1848344	0.332361
Down	DYNC1I1	41	0.009986	4398264	0.2816
Down	UGP2	39	0.010629	2140698	0.318654
Down	KRT5	39	0.006255	1335540	0.352742
Down	THBS1	37	0.010831	751230	0.351315
Down	SULT1A1	36	0.010429	1425372	0.3207
Down	ITGAV	34	0.009907	738818	0.348595
Down	BLNK	34	0.006063	3165890	0.267709
Down	KRT16	34	0.004495	1291100	0.352231
Down	TUBB2B	34	0.006677	1049214	0.327997
Down	TPM2	31	0.005911	1029436	0.321832
Down	SERPINE1	31	0.006739	1233976	0.3258
Down	CSF1R	30	0.006717	1300016	0.330974
Down	TEC	29	0.006824	858984	0.329152
Down	UGDH	28	0.009574	1182896	0.320334
Down	EEA1	28	0.00918	1154334	0.31573
Down	CYBB	27	0.007148	1130954	0.315922
Down	SNCAIP	25	0.006301	1035622	0.319856
Down	HEXIM1	25	0.006057	1136892	0.320587
Down	PSAT1	24	0.005926	1376392	0.324034
Down	CASP6	23	0.005143	904188	0.326939
Down	CCND2	23	0.004537	916146	0.317683
Down	TFAP2C	23	0.0052	855498	0.321095
Down	C1QA	23	0.006243	387922	0.312967
Down	PDLIM1	22	0.005357	928252	0.314967
Down	FAM118B	22	0.008601	929084	0.314885
Down	MARCKS	22	0.00416	786398	0.318099
Down	KLF10	21	0.005139	897850	0.31669
Down	DACT1	20	0.00252	987976	0.277507
Down	LBP	20	0.004692	404246	0.288597
Down	AP1M2	20	0.005509	738138	0.318989
Down	ETV1	20	0.00302	609754	0.314315
Down	CACNA1C	19	0.004425	753630	0.318849
Down	F2R	19	0.005228	757806	0.314586
Down	WWC1	19	0.004648	1378328	0.261415
Down	SPARC	19	0.002419	271042	0.288871
Down	ETV5	19	0.006356	766338	0.321548
Down	HLA-DRB1	18	0.004301	753340	0.315922
Down	CD2	18	0.004823	668514	0.31658
Down	GTF2A1L	18	0.007186	2863798	0.23688
Down	GEM	17	0.005732	2326034	0.240583
Down	GNG2	16	0.003555	589398	0.314126
Down	IKBIP	16	0.004022	727254	0.319017
Down	METTL21C	15	0.003787	493576	0.315621
Down	ANKRD1	15	0.003619	472178	0.313774
Down	PTPRZ1	15	0.001661	140480	0.283902
Down	TRPV4	15	0.002549	481158	0.316662
Down	KCNA2	15	0.00436	1061814	0.239903
Down	ITGB1BP2	14	0.001989	240810	0.285502
Down	FSTL1	14	0.00427	2031070	0.23759
Down	CYP2C9	14	0.001662	302482	0.26514
Down	MTHFD1L	14	0.002585	651008	0.31647
Down	IQGAP2	13	0.002809	325554	0.314045
Down	PAG1	13	0.001736	135246	0.277571
Down	F13A1	13	0.001735	158432	0.30128
Down	FCGR2B	13	0.001066	801788	0.251639
Down	PALLD	13	0.003467	531744	0.313532
Down	CD28	13	0.00323	284756	0.32323
Down	RABIF	13	0.004967	429618	0.312832
Down	GRB14	12	0.002983	685690	0.249385
Down	LIMCH1	12	0.003625	2733430	0.254786
Down	FBN1	11	0.002676	383518	0.248755
Down	SAMSN1	11	8.53E-04	133970	0.288871
Down	EPHB3	11	0.003278	542624	0.316607
Down	ENC1	11	0.001583	423908	0.317324
Down	TRIM50	11	0.00292	496854	0.312886
Down	SERPINE2	11	0.002799	2273346	0.242664
Down	HLA-DQA1	11	0.002544	326930	0.314369
Down	ARRDC4	10	0.001535	375436	0.315157
Down	RND3	10	0.00448	330498	0.313182
Down	VLDLR	10	0.004556	320522	0.314804
Down	SOCS5	10	0.003457	380282	0.320869
Down	BAMBI	10	0.004199	323112	0.313909
Down	NAIP	9	0.003621	141406	0.276246
Down	DRD1	9	0.003299	1100044	0.229184
Down	GLB1	9	0.002193	292422	0.314858
Down	HGF	9	0.001854	1215984	0.238226
Down	ALX4	9	0.003365	1011538	0.2046
Down	COL3A1	9	5.70E-04	68058	0.284833
Down	MTF1	9	0.002252	733044	0.272103
Down	LOX	8	0.001541	584690	0.240345
Down	CACNG2	8	0.002377	668336	0.245242
Down	ANGPT2	8	8.97E-04	173688	0.314641
Down	INHBA	8	0.001672	81898	0.210214
Down	CCDC136	8	9.01E-04	360770	0.220509
Down	CCL18	8	0.001487	441826	0.255876
Down	PMEPA1	7	5.77E-04	135816	0.312967
Down	STAB1	7	0.001896	241374	0.264544
Down	KCNA1	7	6.54E-04	95830	0.211727
Down	CRABP2	7	4.03E-04	99166	0.313209
Down	PTGFRN	7	0.002199	667760	0.225681
Down	ASAH1	7	0.001266	221440	0.315457
Down	RCN3	7	0.00299	297670	0.314072
Down	FRZB	7	0.002208	481180	0.22015
Down	SLIT2	6	0.001658	181082	0.312967
Down	CLEC4M	6	0.001712	388848	0.245111
Down	TNFRSF12A	6	0.001027	142760	0.312671
Down	INSM1	6	0.001234	305024	0.249044
Down	SFRP2	6	6.16E-04	195012	0.229515
Down	SESN1	6	4.86E-04	138422	0.313801
Down	JAZF1	5	2.77E-04	135572	0.315922
Down	LINGO1	5	0.001129	296570	0.207901
Down	SLC5A1	5	5.97E-04	499752	0.26091
Down	SERF1A	5	4.54E-04	225474	0.3168
Down	TLR7	5	6.25E-04	167802	0.209634
Down	AMPD1	5	5.58E-04	409250	0.245374
Down	HPD	5	7.74E-04	98924	0.313936
Down	ICAM5	1	0	0	0.244667
Down	ADRA1B	1	0	0	0.262695

### Target gene – miRNA regulatory network construction and analysis

**Fig. 4:**
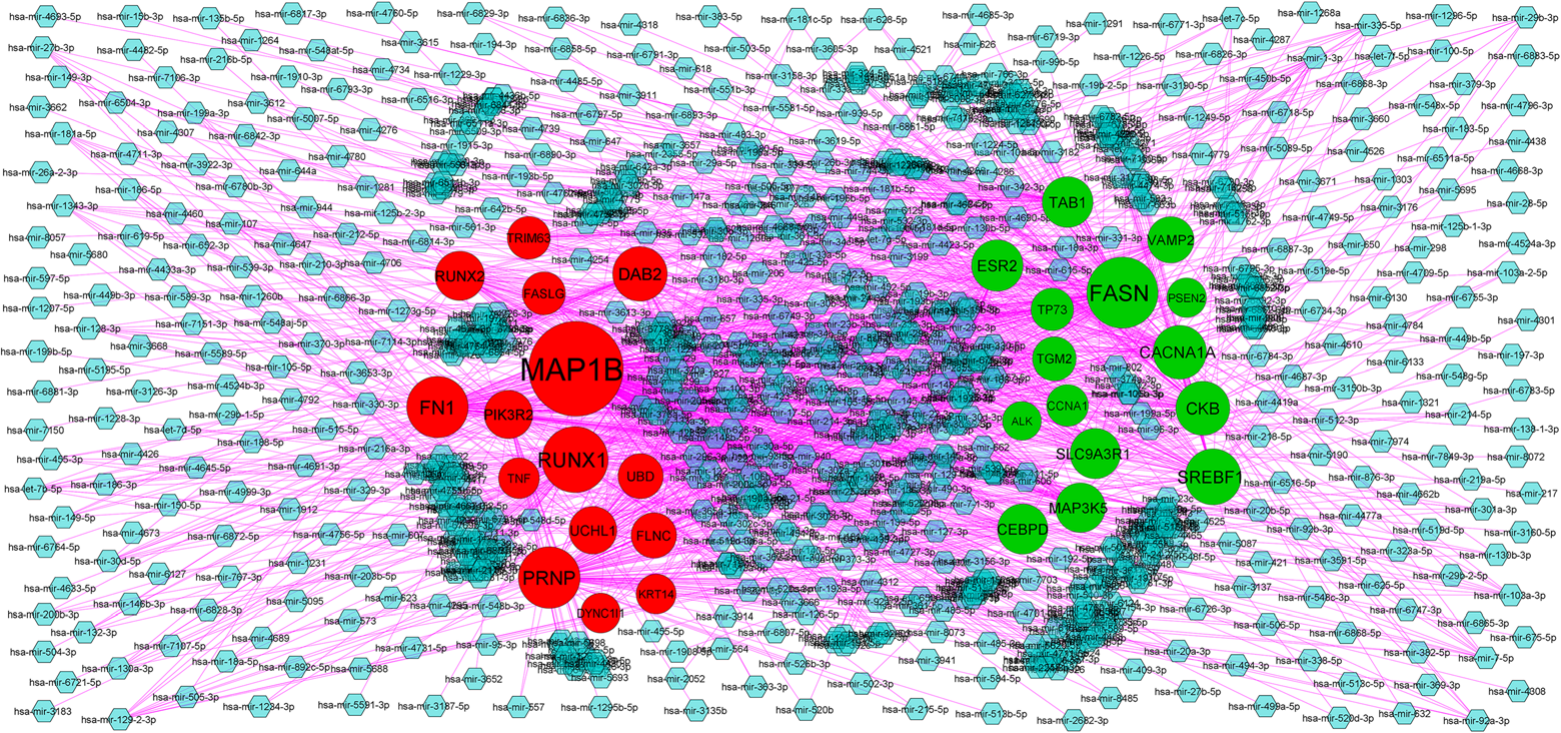
Target gene - miRNA regulatory network between target genes. The blue color diamond nodes represent the key miRNAs; up regulated genes are marked in green; down regulated genes are marked in red

The target genes - miRNA regulatory network was constructed, including 1982 miRNAs and 245 target genes. As shown in the integrated target genes - miRNA regulatory network (Fig.4), FASN targeted 147 miRNAs (ex, hsa-mir-4314), SREBF1 targeted 81 miRNAs (ex, hsa-mir-5688), CKB targeted 72 miRNAs (ex, hsa-mir-583), CACNA1A targeted 69 miRNAs (ex, hsa-mir-632), ESR2 targeted 61 miRNAs (ex, hsa-mir-3176), MAP 1B targeted 249 miRNAs (ex, hsa-mir-1299), RUNX1 targeted 125 miRNAs (ex, hsa-mir-4530), PRNP targeted 106 miRNAs (ex, hsa-mir-4477a), FN1 targeted 105 miRNAs (ex, hsa-mir-606) and DAB2 targeted 75 miRNAs (ex, hsa-mir-1343-3p6) and are listed in Table 6.

**Table 6:** miRNA - target gene and TF - target gene interaction

Regulation	Target Genes	Degree	MicroRNA	Regulation	Target Genes	Degree	TF
Up	FASN	147	hsa-mir-4314	Up	SREBF1	94	ATF4
Up	SREBF1	81	hsa-mir-5688	Up	FASN	71	CUX1
Up	CKB	72	hsa-mir-583	Up	SLC9A3R1	63	MBD2
Up	CACNA1A	69	hsa-mir-632	Up	CKB	50	IRF4
Up	ESR2	61	hsa-mir-3176	Up	TGM2	50	SIN3A
Up	TAB1	58	hsa-mir-4438	Up	VAMP2	32	GABPA
Up	SLC9A3R1	56	hsa-mir-6133	Up	PSEN2	16	TGIF2
Up	CEBPD	56	hsa-mir-4433a-3p	Up	CEBPD	15	SAP30
Up	MAP 3K5	53	hsa-mir-4753-3p	Up	TP73	11	WRNIP1
Up	VAMP2	39	**hsa-mir-2355-5p**	Up	TAB1	6	KLF9
Up	TGM2	27	hsa-mir-375	Up	CCNA1	3	SUPT5H
Up	TP73	22	hsa-mir-7114-3p	Up	ESR2	1	IRF1
Up	CCNA1	19	hsa-mir-7-5p	Down	PIK3R2	73	ZNF143
Up	PSEN2	9	hsa-mir-29b-2-5p	Down	FLNC	53	SMARCE1
Up	ALK	6	hsa-mir-132-3p	Down	RUNX1	53	ZBTB7A
Down	MAP 1B	249	hsa-mir-1299	Down	FN1	45	CREB1
Down	RUNX1	125	hsa-mir-4530	Down	TRIM63	22	RELA
Down	PRNP	106	hsa-mir-4477a	Down	TNF	20	NFIC
Down	FN1	105	hsa-mir-606	Down	PRNP	19	KLF11
Down	DAB2	75	hsa-mir-1343-3p	Down	MAP 1B	13	CREM
Down	RUNX2	52	hsa-mir-944	Down	FASLG	5	BACH1
Down	PIK3R2	48	hsa-mir-1912	Down	UBD	3	TEAD3
Down	UCHL1	45	hsa-mir-20a-3p	Down	DYNC1I1	2	ZNF71
Down	FLNC	35	hsa-mir-455-3p	Down	UCHL1	2	ZNF610
Down	UBD	34	hsa-mir-214-5p	Down	RUNX2	1	NR2F6
Down	FASLG	25	hsa-mir-7849-3p				
Down	TRIM63	24	hsa-mir-3660				
Down	TNF	15	hsa-mir-130a-3p				
Down	KRT14	11	hsa-mir-1343-3p				
Down	DYNC1I1	9	hsa-mir-129-2-3p				

### Target gene-TF s regulatory network construction and analysis

**Fig. 5:**
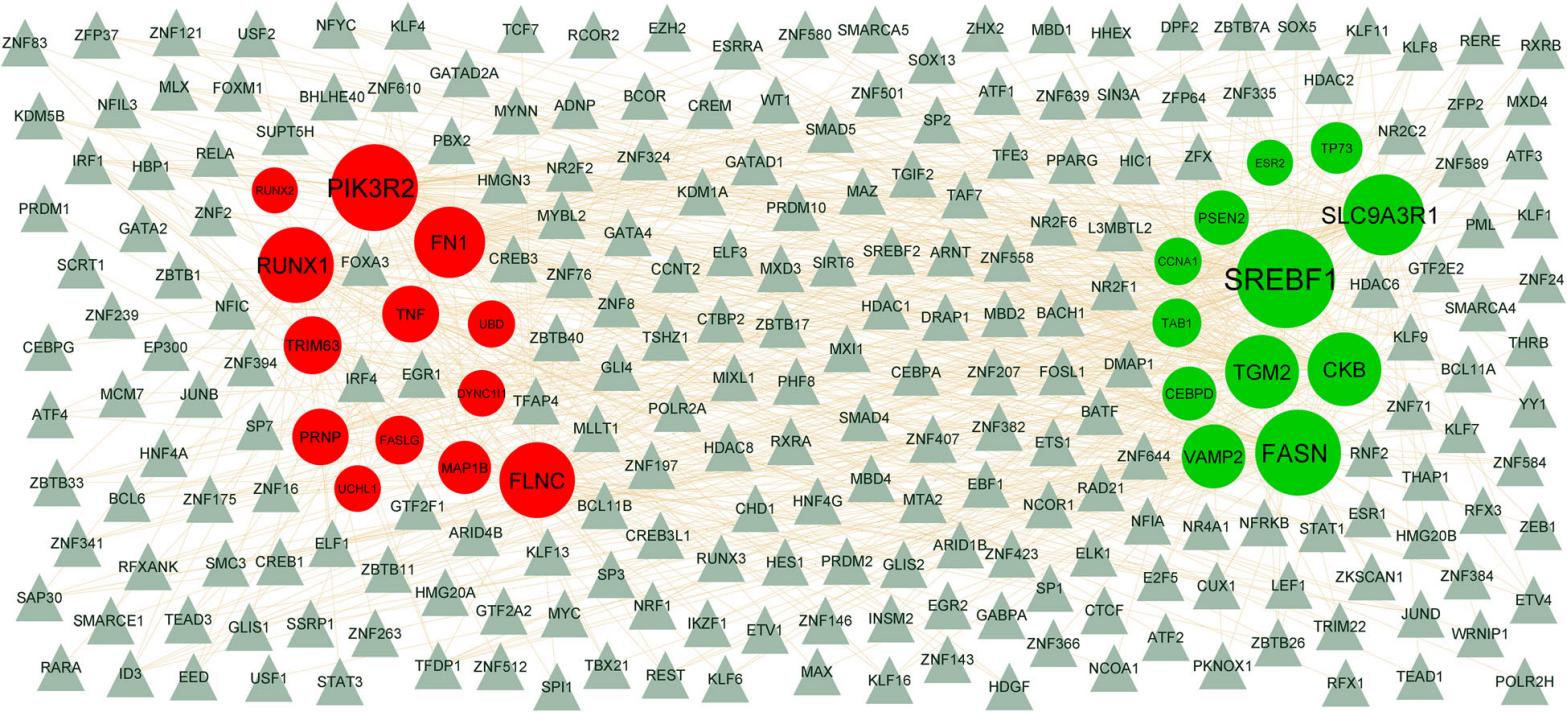
Target gene - TF regulatory network between target genes. The gray color triangle nodes represent the key TFs; up regulated genes are marked in green; down regulated genes are marked in red

The target genes -TF regulatory network was constructed, including 333 TFs and 204 target genes. As shown in the integrated target genes -TF regulatory network (Fig. 5), SREBF1 targeted 94 TFs (ex, ATF4), FASN targeted 71 TFs (ex, CUX1), SLC9A3R1 targeted 63 TFs (ex, MBD2), CKB targeted 50 TFs (ex, IRF4), TGM2 targeted 50 TFs (ex, SIN3A), PIK3R2 targeted 73 TFs (ex, ZNF143), FLNC targeted 53 TFs (ex, SMARCE1), RUNX1 targeted 53 TFs (ex, ZBTB7A), FN1 targeted 45 TFs (ex, CREB1) and TRIM63 targeted 22 TFs (ex, RELA) and are listed in Table 6.

### Receiver operating characteristic (ROC) curve analysis

**Fig. 6:**
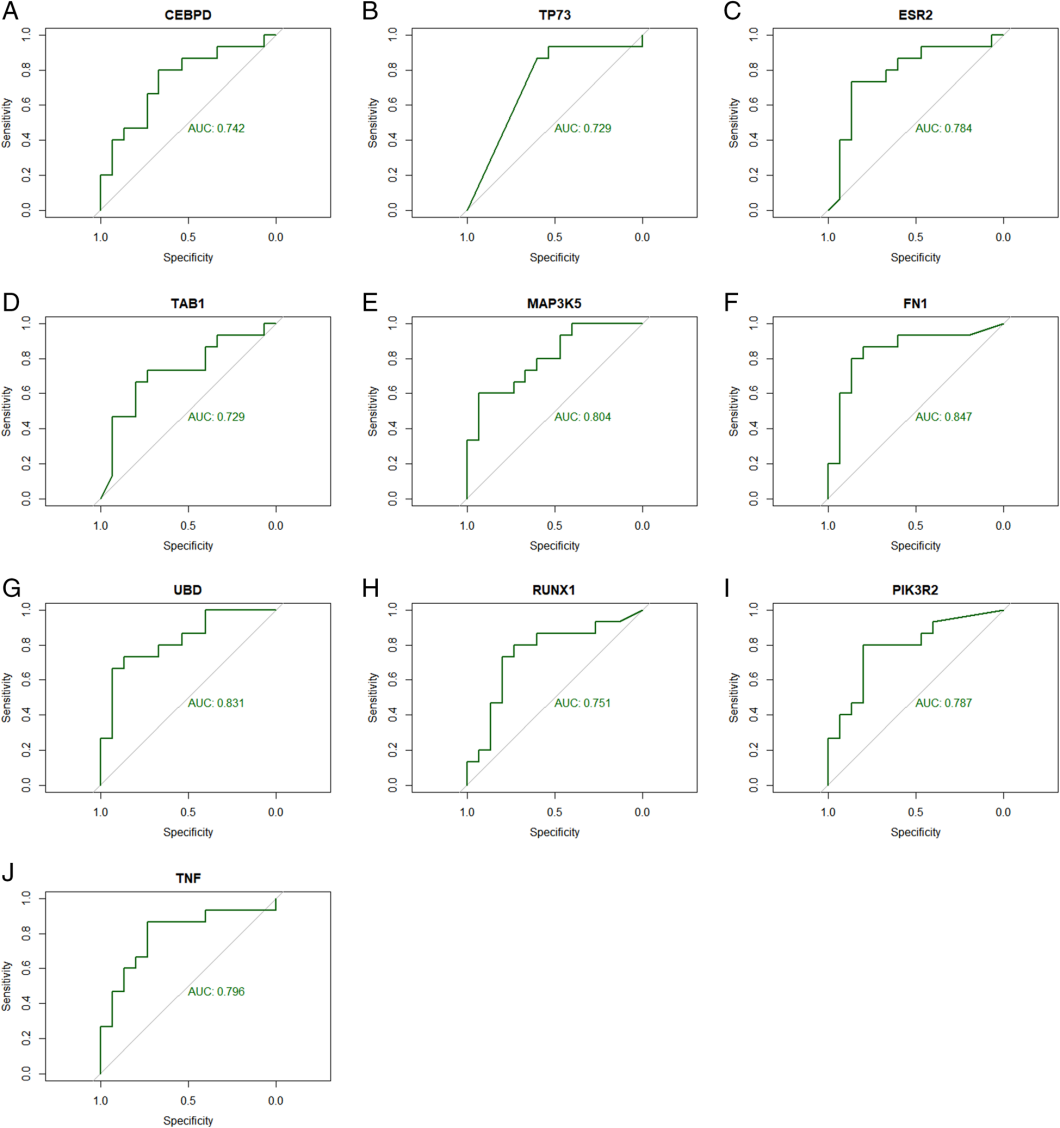
ROC curve analyses of hub genes. **a** CEBPD **b** TP73 **c** ESR2 **d** TAB1 **e** MAP 3K5 **f** FN1 **g** UBD h RUNX1 **i** PIK3R2 **j** TNF

The ROC curve analysis was used to assess the predictive accuracy of hub genes. AUC was determined and used to prefer the most appropriate cut-off gene expression levels. ROC curves and AUC values are presented in Fig. 6. All AUC values exceeded 0.72, while the up regulated genes CEBPD, TP73, ESR2, TAB1 and MAP 3K5, and down regulated genes FN1, UBD, RUNX1, PIK3R2 and TNF had AUC values > 0.75.

### Validation of the expression levels of candidate genes by RT-PCR

**Fig. 7:**
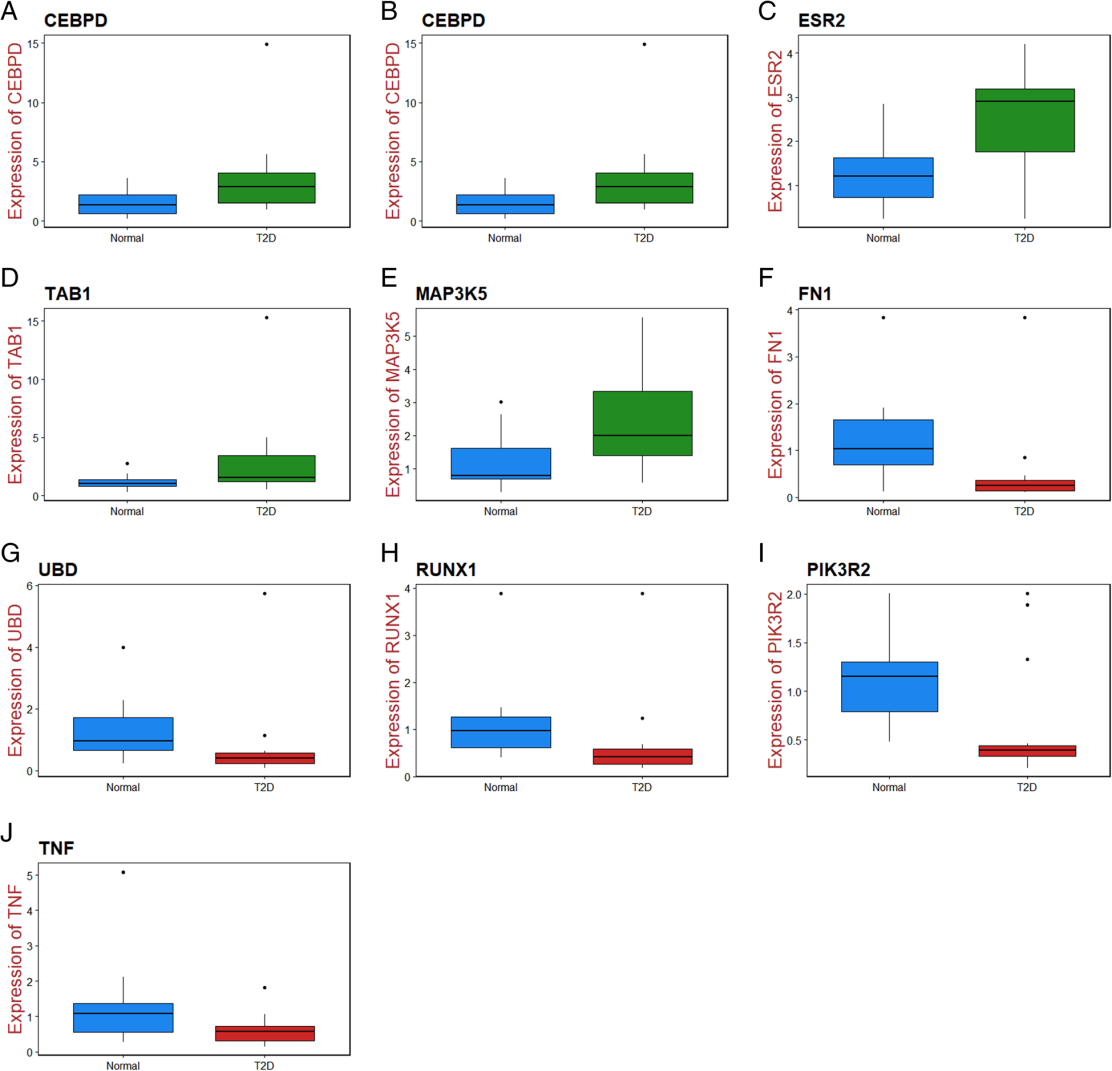
RT-PCR analyses of hub genes. A) CEBPD B) TP73C) ESR2 D) TAB1 E) MAP 3K5 F) FN1 G) UBD H) RUNX1 I) PIK3R2 J) TNF

To further verify the expression level of hub genes in obese samples, RT-PCR was performed to calculate the mRNA levels of the ten hub genes identified in the present study (CEBPD, TP73, ESR2, TAB1, MAP 3K5, FN1, UBD, RUNX1, PIK3R2 and TNF) in obese samples. As illustrated in Fig. 7, the expression of CEBPD, TP73, ESR2, TAB1, MAP 3K5 were significantly up regulated in obese samples compared with normal control tissues, while FN1, UBD, RUNX1, PIK3R2 and TNF were significantly down regulated in obese samples compared with normal control tissues. The present RT-PCR results were in line with the prior bioinformatics analysis, suggesting that these hub genes might be associated with progression of obesity associated type 2 diabetes mellitus.

### Molecular docking studies

**Fig. 8:**
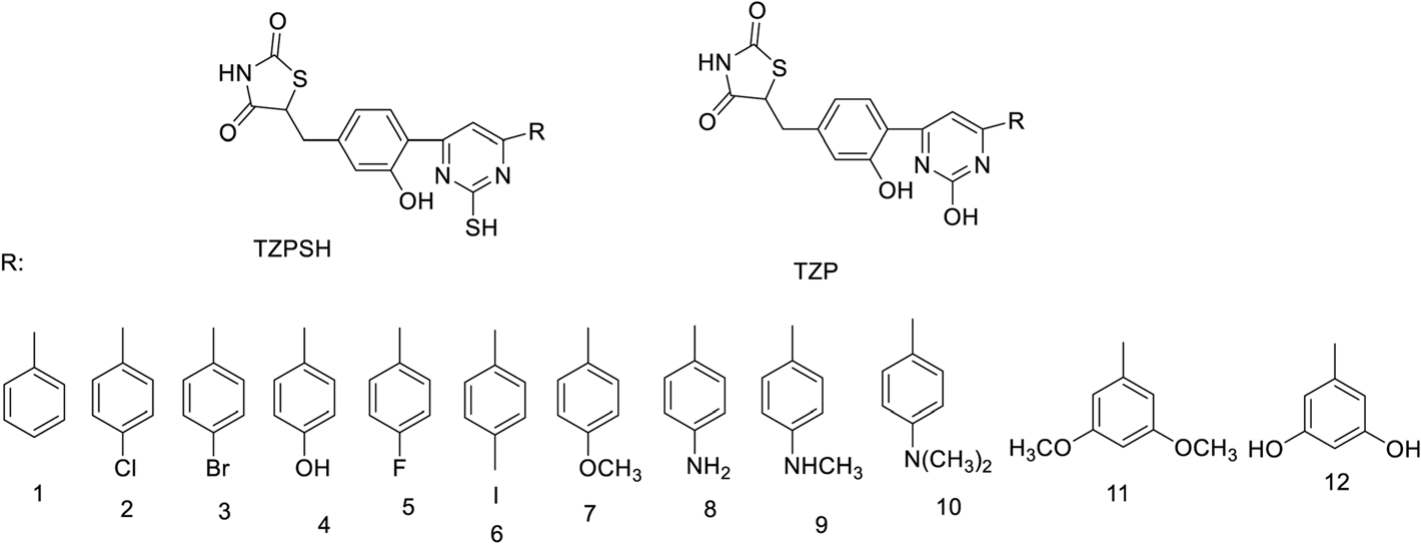
Structures of designed molecules

**Fig. 9:**
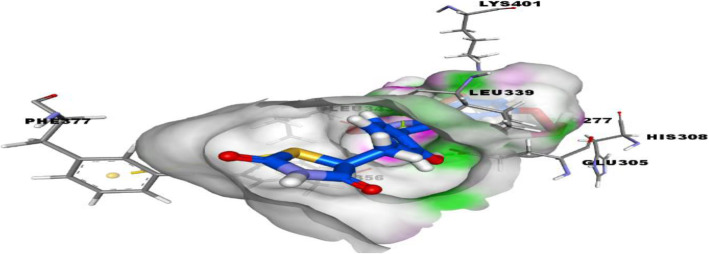
3D Binding of molecule TZP22with 2IOG

**Fig. 10:**
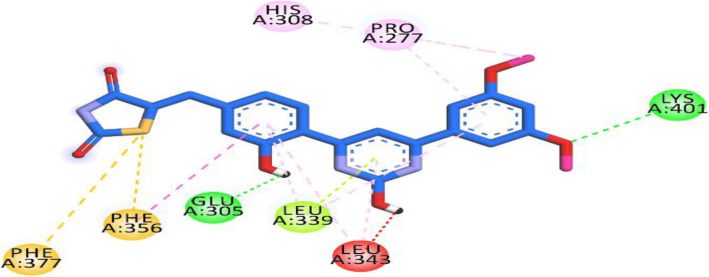
2D Binding of molecule TZP22with 2IOG

In the current research, the docking simulation was conducted to recognize the active site conformation and major interactions responsible for complex stability with the binding sites receptor. Drug design software Sybyl X 2.1 was used to perform docking experiments on novel molecules containing thiazolidindioneheterocyclic ring. Molecules containing the heterocyclic ring of thiazolidinedione are constructed based on the pioglitazone structure and are most widely used alone or in conjunction with other anti-diabetic drugs. Obesity associated type 2 diabetes mellitus is a chronic disorder that prevents insulin from being used by the body the way it should. It's said that people with obesity associated type 2 diabetes mellitus have insulin resistance, oral hypoglycaemic agents are used either alone or in combination of two or more drugs. Pioglitazone (Glitazones) are commonly used either alone or in combination in obesity associated type 2 diabetes mellitus. The one protein in each over expressed genes in obesity associated type 2 diabetes mellitus are selected for docking studies. The X-RAY crystallographic structure of one protein from each over-expressed genes of CEBPD, TP73, ESR2, TAB1 and MAP 3K5, and their co-crystallized PDB code of 4LY9, 2XWC, 2IOG, 5NZZ and 5UP3 respectively were selected for docking. The examinations of the designed molecules were performed to recognize the potential molecule. The foremost of the designed molecules obtained C-score greater than 6 and are said to be active. A total of 24 designed molecules few molecules have excellent good binding energy (C-score) greater than 8 respectively. Few of the designed molecules obtained good binding scores such as molecule TZP20, TZPS8, TZP22, TZPS10 (Fig.8) obtained binding core of 12.212, 11.489, 11.013 and 10.851 with 5UP3 and molecule TZP22, TZPS8, TZPS10 obtained binding score of 9.482, 9.329 and 9.252 with 2XWC and molecule TZP20, TZPS10 obtained binding score 7.359 and 6.848 with 5NZZ and molecule TZP22, TZP21, TZPS9 obtained binding score 11.053, 10.716 and 10.669 with 2IOG respectively. The molecule TZP23, TZPS5, TZPS2 obtained bind score 4.336 to 4.319 with 5NZZ and molecule TZPS10 of binding core 4.633 with 2IOG respectively. The binding score of the predicted molecules are compared with that of the standard pioglitaone obtained bind score of 10.1314, 9.834, 9.8244, 9.8284 and 7.4321 with 2IOG, 2XWC, 4LY9, 5UP3 and 5NZZ, the values are depicted in Table 7. The molecule TZP22 obtained good binding score with all proteins and hydrogen bonding and other bonding interactions with amino acids with protein code 2IOG are depicted by 3D (Fig.9) and 2D (Fig.10) figures.

**Table 7:** Docking results of designed molecules on over expressed proteins

Sl. No/ Code	CEBPD			TP73			ESR2			TAB1			MAP 3K5		
	PDB: 4LY9			PDB:2XWC			PDB: 2IOG			PDB: 5NZZ			PDB: 5UP3		
	Total Score	Crash (-Ve)	Polar	Total Score	Crash (-Ve)	Polar	Total Score	Crash (-Ve)	Polar	Total Score	Crash (-Ve)	Polar	Total Score	Crash (-Ve)	Polar
TZPS1	6.566	-2.405	3.689	7.9803	-1.081	5.097	7.0797	-2.8787	1.281	5.0431	-0.6948	4.499	6.5667	-2.4057	3.689
TZPS2	6.800	-1.167	3.419	6.1601	-1.083	6.357	7.8372	-2.6932	2.462	4.6726	-1.1262	1.907	6.8009	-1.1671	3.419
TZPS3	6.468	-1.228	3.868	6.0227	-1.072	5.427	7.3618	-3.0167	1.176	4.8565	-1.1463	2.469	6.4686	-1.2285	3.868
TZPS4	7.715	-1.553	4.079	6.9313	-0.970	3.418	6.9469	-3.4753	1.281	5.5469	-1.4023	3.550	7.7156	-1.5531	4.079
TZPS5	6.453	-2.306	3.410	7.6894	-0.931	4.526	8.4667	-3.7554	4.692	4.3198	-1.0241	1.117	6.4536	-2.3064	3.410
TZPS6	6.461	-1.855	3.560	6.4527	-0.984	4.601	7.4944	-2.9796	1.224	4.9436	-1.2781	2.196	6.4616	-1.8552	3.560
TZPS7	8.534	-0.828	5.290	7.0007	-0.724	4.207	7.5843	-2.8172	1.999	4.7368	-1.3057	1.168	8.5349	-0.8288	5.290
TZPS8	11.489	-2.122	7.200	9.3291	-0.883	7.126	8.2146	-3.2986	3.067	6.7141	-0.8055	4.503	11.4898	-2.1229	7.200
TZPS9	7.152	-2.611	2.884	6.6449	-1.505	3.473	10.6699	-4.5053	5.965	5.0364	-1.7434	2.868	7.1521	-2.6116	2.884
TZPS10	10.851	-1.162	8.210	9.2527	-2.282	9.981	4.6334	-5.6706	3.280	6.8487	-1.4021	5.550	10.8518	-1.1628	8.210
TZPS11	7.549	-1.776	5.977	7.7814	-0.869	6.651	6.5663	-3.9855	2.880	5.2886	-1.4319	2.135	7.549	-1.7765	5.977
TZPS12	6.421	-1.515	3.412	5.2317	-1.441	5.132	7.4944	-2.9796	1.224	4.9436	-1.2781	2.196	6.4616	-1.8552	3.560
TZP13	7.906	-2.434	4.218	7.2083	-0.547	5.237	8.0517	-2.3234	2.222	4.697	-1.0273	2.204	7.9062	-2.4344	4.218
TZP14	7.674	-3.098	4.391	7.9457	-0.894	5.848	6.2749	-3.9247	1.839	4.7119	-0.9488	1.085	7.6749	-3.0982	4.391
TZP15	7.183	-2.936	3.636	7.1064	-0.980	4.143	8.5779	-3.6225	2.525	5.0291	-0.944	2.222	7.1835	-2.9367	3.636
TZP16	6.406	-1.533	1.860	6.3759	-0.991	3.531	8.2194	-1.9538	2.196	4.8598	-0.9268	3.062	6.4065	-1.5333	1.860
TZP17	8.690	-2.478	5.875	6.6083	-1.099	3.064	8.6505	-2.9891	3.094	4.948	-1.1025	3.928	8.6902	-2.4782	5.875
TZP18	6.770	-1.638	3.659	6.8998	-0.879	4.661	7.4019	-3.3357	2.698	5.0051	-1.0824	2.177	6.7708	-1.6383	3.659
TZP19	8.628	-1.406	5.189	8.3366	-1.139	5.462	8.8114	-4.448	5.183	4.7401	-1.8841	2.637	8.6283	-1.4062	5.189
TZP20	12.212	-2.219	8.540	8.9043	-0.806	6.430	10.7167	-2.127	5.792	7.3594	-1.4906	5.170	12.2126	-2.2192	8.540
TZP21	8.030	-1.557	5.434	8.2243	-1.099	6.851	8.2053	-4.0771	1.467	5.207	-1.2243	3.859	8.0309	-1.5573	5.434
TZP22	11.013	-2.175	8.175	9.4828	-0.737	9.663	11.0511	-5.3734	6.934	5.7841	-0.7944	5.493	11.0134	-2.175	8.175
TZP23	7.965	-3.168	6.847	8.5304	-1.107	7.803	8.0344	-2.5889	4.518	4.3368	-1.305	2.127	7.9652	-3.1688	6.847
TZP24	6.770	-1.638	3.659	6.8998	-0.879	4.661	7.4019	-3.3357	2.698	5.0051	-1.0824	2.177	6.7708	-1.6383	3.659
Standard Pioglitazone	9.8244	-2.396	5.097	9.834	-0.633	8.476	10.1314	-1.7567	2.8556	7.4321	-0.7906	4.505	9.8284	-2.396	5.097

## Discussion

Obesity associated type 2 diabetes mellitus is the most common aggressive metabolic disorder [37]. However, the most key challenge in treating obesity associated type 2 diabetes mellitus is the presence of complexity [38]. Although previous investigations have reported various potential molecular markers linked with the advancement of obesity associated type 2 diabetes mellitus, the molecular mechanism underlying its pathogenesis has not been generally studied [39]. In the present investigation, a total of 820 DEGs were identified, containing 409 up regulated genes and 411 down regulated genes. SULT1C2 [40] and UBD (ubiquitin D) [41] were responsible for progression of kidney diseases, but these genes might be liable for advancement of obesity associated type 2 diabetes mellitus. HLA-DQA1 was associated with progression of T2DM [42]. SPX (spexin hormone) [43] and APOB (apolipoprotein B) [44] are a critical proteins plays an important role in obesity associated type 2 diabetes mellitus.

The GO and pathway enrichment analysis of DEG are closely related to obesity associated type 2 diabetes mellitus. Genes such as KCNE5 [45], SHANK3 [46], CASQ2 [47], EDNRA (endothelin receptor type A) [48], EPHB4 [49], ALPK3 [50], WNT11 [51], IRAK2 [52], FBN1 [53], SFRP2 [54], CLCA2 [55], NEXN (nexilin F-actin binding protein) [56], PALLD (palladin, cytoskeletal associated protein) [57], DAB2 [58], NRP2 [59], THBS2 [60], CSF1R [61], KCNA2 [62], CACNA1C [63], F2R [64], UCHL1 [65], CCL18 [66], ITGB1BP2 [67] and FMOD (fibromodulin) [68] were reportedly involved in cardio vascular diseases, but these genes might be key for progression of obesity associated type 2 diabetes mellitus. Hu et al. [69], Liu et al. [70], Eltokhi et al. [71], Cai et al. [72], Pfeiffer et al. [73], Lin et al. [74], Royer-Zemmour et al. [75], Pastor et al. [76], Goodspeed et al. [77], Zhang et al. [78], Rogers et al. [79], Su et al. [80] and Foale et al. [81] reported that NRXN1, CRHR1, SHANK2, PSEN2, CKB (creatine kinase B), CD200R1, SRPX2, PTPRZ1, SLC6A1, GABRB2, KCNA1, ASAH1 and LINGO1 were the genes expressed in progression of neuropsychiatric disorders, but these genes might be involved in advancement of obesity associated type 2 diabetes mellitus. Reports indicate that genes include SPHK2 [82], NPC1L1 [83], CNTFR (ciliaryneurotrophic factor receptor) [84], SLC2A4 [85], EDA (ectodysplasin A) [86], TGM2 [87], GCK (glucokinase) [88], FASN (fatty acid synthase) [89], FAP (fibroblast activation protein alpha) [90], PRNP (prion protein) [91], LYVE1 [92], SERPINE1 [93], TNF (tumor necrosis factor) [94], FASLG (Fas ligand) [95], HGF (hepatocyte growth factor) [96], FNDC5 [97], LBP (lipopolysaccharide binding protein) [98] and LOX (lysyl oxidase) [99] were the genes expressed in obesity associated type 2 diabetes mellitus. Hirai et al [100], Vuori et al [101], Porta et al [102], Nomoto et al [103] and Blindbæk et al [104] demonstrates that VAMP2, CACNB2, SLC19A3, PFKFB3 and MFAP4 were the genes essential for progression of type 1 diabetes, but these genes might be key for advancement of obesity associated type 2 diabetes mellitus. Genes such as CACNA1A [105], ALK (ALK receptor tyrosine kinase) [106], SLC4A4 [107], STOX1 [108], COL3A1 [109], VNN1 [110], SLC4A7 [111], BDKRB2 [112], DRD1 [113] and LPAR1 [114] have reported significantly linked with hypertension, but these genes might be crucial for progression of obesity associated type 2 diabetes mellitus. Genes such as KCNE2 [115], DLL1 [116], ACVR1C [117], RGS3 [118], MLXIPL (MLX interacting protein like) [119], PAG1 [120], SLC2A10 [121] and GRB14 [122] play important role in type 2 diabetes mellitus progression. A recent investigation has indicated that genes such as GPIHBP1 [123], FGFRL1 [124], DAPK2 [125], MAP 3K5 [126], ANKK1 [127], GK (glycerol kinase) [128], SPHK1 [129], GNG3 [130], FSTL3 [131], SLIT2 [132], CCDC80 [133], RND3 [134], PTGER4 [135], RUNX1 [136], ADAM12 [137], OLR1 [138], THBS1 [139], CD28 [140], TRPV4 [141], ATRN (attractin) [142], MRC1 [143], SEMA3C [144], HTR2B [145], NOX4 [146], TACR1 [147], BAMBI [148], PDGFD (platelet derived growth factor D) [149], APLN (apelin) [150], MFAP5 [151] and LUM (lumican) [152] are associated with a development of obesity. A previous investigation found that genes such asDDR1 [153], TAB1 [154], NEK8 [155], SERPINE2 [156], FCGR2B [157], ANGPT2 [158], FN1 [159], SOCS5 [158], SMOC2 [160], CD2 [161] and SCN9A [162] expression were associated with a kidney diseases, but these genes might be responsible for advancement of obesity associated type 2 diabetes mellitus.

In addition, an investigation reported that hub genes serve an essential role in maintaining the entire PPI network and its modules are indispensable. 10 hub genes, including CEBPD, TP73, ESR2, TAB1, MAP 3K5, FN1, UBD, RUNX1, PIK3R2 and TNF, were identified as the key genes responsible for progression of obesity associated type 2 diabetes mellitus. Investigation has demonstrated that CEBPD (CCAAT enhancer binding protein delta) is involved in obesity [163]. An investigation by Domingues-Montanari et al. [164] demonstrated that key gene ESR2 was involved in the progression of cardio vascular disease, but this gene might be responsible for progression of obesity associated type 2 diabetes mellitus. TP73, PIK3R2, SLC9A3R1, KRT5, KRT14 and TFAP2C are novel biomarkers for pathogenesis of obesity associated type 2 diabetes mellitus.

The miRNA-target gene regulatory network and TF-target gene regulatory network highlighted in the current investigation provides new theoretical guidance for further exploring the molecular mechanism of obesity associated type 2 diabetes mellitus and provides a new perspective for understanding the underlying biological processes of this diseases, and miRNA and TF targeted therapy. Eberlé et al [165], Cheng et al [166], Cavallari et al [167], Qi et al [168] and Yan et al [169] indicated that SREBF1, MBD2, IRF4, CREB1 and RELA (Nuclear factor-kB) were the genes responsible for advancement of obesity associated type 2 diabetes mellitus. Matsha et al [170] and Ding et al [171] demonstrated that hsa-mir-1299 and hsa-mir-4530 were the miRNAs liable for progression of type 2 diabetes mellitus. Hall et al [172] and Salazar-Mendiguchía et al [173] reported that FLNC (filamin C) and TRIM63 were the genes involved in progression of cardio vascular disease, but these genes might be essential for development of obesity associated type 2 diabetes mellitus. Xiao et al [174], Stratigopoulos et al [175] and Zhou et al [176] noted that ATF4, CUX1 and ZBTB7A were the genes responsible for advancement of obesity. MAP 1B, hsa-mir-4314, hsa-mir-5688, hsa-mir-583, hsa-mir-632, hsa-mir-3176, hsa-mir-4477a, hsa-mir-606, hsa-mir-1343-3p6, SIN3A, ZNF143 and SMARCE1 are the novel biomarkers for pathogenesis of obesity associated type 2 diabetes mellitus.

However, this investigation had distinct limitations. First, the mechanisms of several hub genes in the pathological process of obesity associated type 2 diabetes mellitus remain unclear, permit further investigation. Moreover, the potency of our small molecule drug screening in diminishing side effects remains to be assessed.

In conclusion, with the integrated bioinformatics analysis for expression profiling by high throughput sequencing in obesity associated type 2 diabetes mellitus, ten hub genes associated with the pathogenesis and prognosis of obesity associated type 2 diabetes, including CEBPD, TP73, ESR2, TAB1, MAP 3K5, FN1, UBD, RUNX1, PIK3R2 and TNF. These hub genes were associated with progression of obesity associated type 2 diabetes mellitus and first five (CEBPD, TP73, ESR2, TAB1 and MAP 3K5) of them might be linked with targeted therapy. These hub genes might be regarded as new diagnostic and prognostic biomarkers for obesity associated type 2 diabetes mellitus. However, further in-depth investigation (in vivo and in vitro experiment) is necessary to elucidate the biological function of these genes in obesity associated type 2 diabetes mellitus.

## Data Availability

The datasets supporting the conclusions of this article are available in the GEO (Gene Expression Omnibus) (https://www.ncbi.nlm.nih.gov/geo/) repository. [(GSE143319) (https://www.ncbi.nlm.nih.gov/geo/query/acc.cgi?acc=GSE143319]
